# Prediction of secondary metabolites in hydroponically produced tomatoes: multivariate influence of abiotic climatic factors as well as photosynthesis and transpiration rates

**DOI:** 10.3389/fpls.2025.1543699

**Published:** 2025-02-26

**Authors:** Grigorij Devadze, Dennis Dannehl, Annika Nerlich, Uwe Schmidt, Stefan Streif

**Affiliations:** ^1^ Automatic Control and System Dynamics, Chemnitz University of Technology, Chemnitz, Germany; ^2^ Humboldt-Universität zu Berlin, Albrecht Daniel Thaer-Institute of Agricultural and Horticultural Sciences, Division Biosystems Engineering, Berlin, Germany; ^3^ Robert Koch Institute, Coordination Centre for Quality Management, Berlin, Germany; ^4^ Fraunhofer Institute for Molecular Biology and Applied Ecology, Department of Bioresources, Giessen, Germany

**Keywords:** greenhouse, classification, data augmentation, relative humidity, temperature, CO_2_, light intensity, secondary metabolites

## Abstract

This is the first study who presents an approach to predict secondary metabolites content in tomatoes using multivariate time series classification of greenhouse sensor data, which includes climatic conditions as well as photosynthesis and transpiration rates. The aim was to find the necessary conditions in a greenhouse to determine the maximum content of secondary metabolites, as higher levels in fruits can promote human health. For this, we defined multiple classification tasks and derived suitable classification function. Cross-validated high accuracy results demonstrate the effectiveness of the approach. Considering a period of three weeks, we found that PPFD levels between 396.0 μmol/m^2^s and 511.2 μmol/m^2^s as well as transpiration rates ranging from 4.4 mg H_2_O/m^2^s to 7.47 mg H_2_O/m^2^s were observed as optimal for the contents of beta carotene, lutein, and lycopene. Optimal contents for naringenin and phloretin diglucoside can be achieved at lower PPFD ranges from 229.4 μmol/m^2^s to 431.2 μmol/m^2^s and from 35.76 μmol/m^2^s to 262.28 μmol/m^2^s and at lower transpiration rates from 4.71 to 6.47 mg H_2_O/m^2^s and from 3.04 to 4.26 mg H_2_O/m^2^s, respectively. It was discovered for the first time that, photosynthesis rates also play a significant role in the accumulation of secondary metabolites. Photosynthesis rates between 0.39 μmol CO_2_/m^2^s and 1.21 μmol CO_2_/m^2^s over three weeks were crucial for the optimal accumulation of phenolic acids such as caffeic acid derivates, coumaric acid hexoside, ferulic acid hexoside and coumaroylquinic acids as well as for quercetin and flavonoid. An optimal temperature range between 20.94 and 21.53°C and a PPFD from 250.0 to 375.2 μmol/m^2^s was classified as beneficial to synthesize these compounds. Optimal light intensity for the total phenolic acids (129.35 - 274.34 μmol/m^2^s) and for the total flavonoids (31.24 - 249.31 μmol/m^2^s), the optimum relative humidity levels are between 83.45 - 91.29% and 87.13 - 91.29%, respectively. Based on these results, this study provides the first evidence that the impact of a single climate factor on secondary metabolites in tomato fruits should not be considered in isolation, but rather, all climatic factors during a growth period must be taken into account to predict the optimal accumulation of individual phenolic compounds and carotenoids in tomatoes. Our results have laid the headstone to help growers target their climate controls to maximize the health-promoting phytochemicals in tomatoes.

## 1 Introduction

Over the next few decades (2021-2040), even under intermediate greenhouse gas emissions scenarios, global temperatures are expected to rise by approximately 1.5 to 2.0*°*C (Ipcc, 2021). This predicted increase in temperature is only the beginning of the dilemma, as this affects not only the plant growing parameter temperature but also other parameters such as the relative humidity, CO_2_-concentration and the photosynthetic photon flux density (PPFD) in greenhouses, depending on the location and the greenhouse control system (Ntinas et al., 2020). The increase in temperature in particular can have many detrimental effects on the plants, e.g., the reduction in photosynthesis, plant development, fruit size, fruit yield and secondary plant compounds, such as carotenoids and phenolic compounds (Dumas et al., 2003; Camejo et al., 2005; Van Der Ploeg and Heuvelink, 2005; Dannehl et al., 2012). The latter are playing an increasingly important role in human nutrition, as their consumption is associated with health-promoting properties, which can reduce the occurrence of human prostate cancer, cardiovascular diseases, cardiac dysfunctions, hypertension and neurodegenerative disorders (Eberhardt et al., 2000; Kotake-Nara et al., 2001; Bagchi et al., 2003; Lambert and Yang, 2003; Boyer and Liu, 2004; Jacob et al., 2008; Saini et al., 2020).

Based on these scientific findings, many research groups have been working for years on the topic of controlled environment agriculture which allows adapting the growing conditions to optimize secondary plant compounds in addition to yields. Basic research has shown, for example, that lycopene and phenolic compounds in tomatoes increased with elevated daily mean temperatures ranging between 18*°*C and 22*°*C (Toor et al., 2006; Krumbein et al., 2012). However, it was reported that the lycopene biosynthesis in tomatoes is almost completely stopped below 12*°*C and above 32*°*C (Dumas et al., 2003; Hernandez et al., 2015; Vijayakumar et al., 2021). While these works agree on the influence of temperature on carotenoids and phenolic compounds in tomatoes, the results in terms of the influence of CO_2_-concentration on the mentioned secondary metabolites are controversial. While Boufeldja et al. (2023) detected a decrease in the total carotenoid and lycopene concentration in tomatoes at an elevated CO_2_-concentration (900 ppm), Krumbein et al. (2012) was unable to detect any changes in the lycopene concentration and Pimenta et al. (2023) found an increase in the lycopene, ß-carotene and lutein concentration at similarly elevated CO_2_-concentrations. It is assumed that the varying results regarding the significant influence of CO_2_ on carotenoids in tomatoes are attributable to the specific tomato variety. In contrast, the results regarding the influence of elevated CO_2_-concentrations on the content of total phenolics in tomatoes are clear. These do not change in dependence on the CO_2_-concentration (Boufeldja et al., 2023; Oliva-Ruiz et al., 2023; Pimenta et al., 2023).

Another very important factor for the accumulation of secondary metabolites in vegetables is not only the light quality but also the photosynthetic photon flux density (Thoma et al., 2020). In general, the contents of lycopene and ß-carotene as well as phenolic compounds, including chlorogenic acid rutin and naringenin in tomatoes increase with increasing PPFD (Wilkens et al., 1996; Slimestad and Verheul, 2005). In detail, Brandt et al. (2006), for instance, were able to increase the lycopene content in tomatoes by 100% by increasing the PPFD from 250 µmol/m^2^s to 380 µmol/m^2^s. However, excessive light intensities during the growing season (mean PPFD = 1215 µmol/m^2^s) can also have negative consequences for the synthesis of carotenoids. Compared to a mean PPFD of 849 µmol/m^2^s during the production cycle, it was found that the contents of lycopene, ß-carotene and lutein were reduced by 40%, 41% and 8%, respectively (Formisano et al., 2021). However, the same scientists were also able to show that the contents of total phenolic acid derivates, total flavonoid derivates, total hydroxycinnamoyl quinic acid derivates and total phenolic compounds increased by 16%, 28%, 108% and 25%, respectively, under the same conditions at the higher light intensity.

The extent to which the relative humidity affects the carotenoids and phenolic compounds in tomato fruits is still largely unclear and currently not extensively investigated. To our knowledge, there is only one publication on the effects of different vapor pressure deficits (VPD) on the carotenoids in tomatoes. In this context, Leonardi et al. (2000) did not analyze the carotenoids, but they showed that the color intensity did not change depending on different levels of the VPD. Therefore, it might be concluded that the carotenoids were not affected. Regarding the influence of VPDs on the phenolic compounds in tomatoes, no studies could be found. If the search is extended to other plants, there is at least one publication in which it was found that the total phenolics in lettuce can be increased at lower levels of relative humidity (VPD = 0.69 kPa versus 1.76 kPa) (Amitrano et al., 2021).

In summary, a major disadvantage of all the studies mentioned is that mainly only one abiotic stress factor was changed, while all other influencing factors were kept constant. In greenhouse production, however, various climate parameters interact with each other, the severity of which depends on the climate zone, but also on the design and climate control strategy of the greenhouses. Specifically, a significant increase in temperature in a conventional greenhouse during the summer months leads to a reduction in relative humidity and CO_2_-concentration, caused by the ventilation opening. In contrast, with a semi-closed greenhouse management approach, the ventilation remains closed for longer periods, resulting in an increase in both temperature and relative humidity, as well as CO_2_-concentration (Schmidt et al., 2008; Dannehl et al., 2012, 2014; Ntinas et al., 2020). Based on these multivariate climatic changes, the scientifically generated insights into the influence of individual climatic factors on secondary metabolites in tomatoes cannot be directly applied to greenhouse production, which is also elegantly described to some extent in the review by Poorter et al. (2016). Therefore, the present study aims to incorporate not only the influence of an individual climatic factor on the secondary metabolites in tomato fruits but all climatic changes throughout a growing period. Our approach seeks to determine the parameters by which individual secondary metabolites can be estimated and the climatic conditions within the greenhouse that must be met to achieve the maximum content of secondary metabolites in tomatoes in order to provide consumers with as much of the health-promoting phytochemicals as possible. This approach will also consider photosynthesis as an influential parameter, as the photosynthesis itself represents the output resulting from the input of various climatic factors and can provide precursors for the synthesis of secondary metabolites. This study is a first, preliminary study presenting this new approach over a cultivation period of one year.

## 2 Materials and methods

### 2.1 Cultivation of tomato plants

Tomato plants (n=480) were grown in a Venlo-type greenhouse (260 m2 net acreage) at Humboldt-Universität zu Berlin, Germany from calendar week (CW) 11 – 47 in 2019. Young tomato plants (Solanum lycopersicum L. cv. Avalantino F1) were grown in small rock wool cubes (100 mm x 100 mm x 65 mm) and ordered at Jungpflanzen Gernert GbR (Albertshofen, Germany). They were delivered at a stage of growth when the first inflorescence was visible and were transferred to rock wool growing bags (Cutilene^®^; Tilburg, The Netherlands), which were used as a component in a closed hydroponic growing system. In this context, tomato plants were cultivated in 12 rows on high channels, each channel equipped with 20 growing backs containing 40 plants. The nutrients were delivered via drip irrigation, which was switched on for 300 seconds after a light summation of 70 klxh was reached. The irrigation time was regularly adjusted to ensure an overflow of 20%. The nutrient solution was mixed according to the recipe of Göhler and Molitor (2002). The target temperature for heating was set at 17°C all day and the ventilation was opened above 23°C to avoid plant damage. The temperature determines the ventilation opening, which opens proportionally to the temperature increase between 0 and 100%. The CO2-concentration in the greenhouse was also controlled, with the aim of maintaining it at 800 ppm during daylight hours. CO2 supply stopped when the ventilation opening exceeded 10% to reduce the CO2 release into the environment. In this context, the temperature and relative humidity in the greenhouse were recorded using a PTF 30 measuring system (PTF 30, Steinbeis GmbH & Co. KG; Stuttgart, Germany). The CO2-concentration in the greenhouse was measured with a high-sensitivity CO2 sensor (CO2 Probe GMP343, Vaisala GmbH, Hamburg, Germany). Photosynthetic photon flux density (PPFD) was measured outside the greenhouse at a height of 8 m using a quantum sensor (LI-190R-BL-2, LI-COR^®^ Biosciences GmbH, Homburg, Germany) and is expressed as µmol/m2s.

To measure photosynthesis and transpiration of tomato plants, the BERMONIS leaf cuvette-based gas exchange system (Steinbeis GmbH & Co. KG; Stuttgart, Germany) was used, where eight leaf cuvettes were attached accordingly to 8 different leaves on several plants. The measurement principle of this device was described in detail by Dannehl et al. (2021).

All mentioned measurements were recorded every 30 seconds from June to November in 2019 by a central computer.

### 2.2 Sample preparation for laboratory analyses

From a plant population of 400 plants, fruits from 45 different plants were randomly harvested according to the Organisation for Economic Co-operation and Development (OECD) color gauge from position one of the fifth truss at ripening stage 10 and divided into three pooled samples of 15 tomatoes each in order to compare three biological replicates. As such, it was ensured the same age of fruits and that no changes in the secondary plant compounds were caused by the influence of different light conditions. This yield procedure war used from 11^st^ June to 25^th^ November at intervals of three weeks.

Shortly thereafter, each tomato was quartered, where two quarter of 15 tomatoes were merged into one sample and immediately frozen in liquid nitrogen and then freeze-dried (Christ Alpha 1-4, Christ; Osterode, Germany) for seven days. These samples were later used to analyze secondary metabolites. The other two quarters were used for the determination of the dry mass using a ventilated oven (Heraeus, Hanau, Germany) kept at 60°C for seven days. The dry matter content of tomatoes was calculated by the ratio of the dry mass to the fresh mass, which is expressed as %.

### 2.3 Determination of phenolic compounds

Freeze-dried tomato fruits were ground to a fine powder (MM 30, Retsch GmbH, Haan, Germany) and stored at -80°C until phenolic acids and flavonoids were analyzed. Extraction and determination of these secondary plant compounds was performed as described by Förster et al. (2015). A high-performance liquid chromatography (HPLC) (Ultimate 3000, Thermo Scientific) equipped with a 150 x 2.1mm C16 column (AcclaimPA, 3 µm, Thermo Scientific) was used as the equipment for the analyses. Commercial standards from Sigma-Aldrich served as references. Peak areas of detected phenolic compounds were used to calculate the contents of each phenolic acid and flavonoid, which were summed up to obtain the total phenolic acid and flavonoid content in tomatoes. The phenolic compounds are expressed as µg/g dry weight (DW).

### 2.4 Determination of carotenoids

The method utilized in this study, with slight modifications as described by Mageney et al. (2016), was employed for the extraction of carotenoids. A freeze-dried tomato powder sample weighing 10 mg was mixed with 500 µL of a methanol-tetrahydrofuran solution (1:1, v/v; extraction solution) and shaken for 5 minutes at a temperature of 24°C and a speed of 500 rpm. Subsequently, the samples were centrifuged at 4,500 rpm for five minutes at a temperature of 20°C, and the resulting supernatant was transferred to a glass vial. The pellet was subjected to two additional extractions using 500 µL of the extraction solution. The collected extracts were then evaporated under a flow of nitrogen until the pellet remained. The pellet was dissolved in 100 µL of dichloromethane and 300 µL of isopropyl alcohol, followed by centrifugation at 3,000 rpm for five minutes. The solution was then filtered using Corning^®^ Costar^®^ Spin-X^®^ centrifuge tube filters (Merck KGaA, Darmstadt, Germany). Subsequently, the filtered extracts were transferred to opaque HPLC vials with inlay.

To analyze the carotenoids, 10 µL of the extraction solution was injected into an Ultimate 3000 HPLC system (Thermo Scientific) equipped with a carotenoid column (YMC-Carotenoid column) and separated at a flow rate of 0.2 mL/min. Detection was performed at a wavelength of 456 nm, and the oven temperature was maintained at 25°C. The eluents consisted of a mixture of methanol, methyl tert-butyl ether, and Milli-Q water (eluent A: 81/15/4, eluent B: 6/90/4). The separation of the carotenoid molecules was achieved using the following gradient: 0-10 min, 0% B; 10-40 min, 0-100% B; 40-42 min, 100% B; 42-45 min, 100-0% B; 45-55 min, 0% B. Commercial standards obtained from Sigma-Aldrich were used as references. For each extraction analysis, a separate injection of 5 µL of a lycopene standard solution (concentration: 1 nmol/µL) was performed, corresponding to a total of 5 nmol. The peak area of the lycopene standard, known for its concentration, along with the determined response factors (RF) for β-carotene (RF = 0.65) and lutein (RF = 0.79) relative to lycopene, were utilized to calculate the content of each identified carotenoid. The individual carotenoid contents were then summed to obtain the total carotenoid content in tomato fruit, expressed as µg/g DW.

### 2.5 Data collection and statistical analysis

The evaluation of the data with regard to the individual secondary metabolites in the tomatoes depending on the harvest dates was carried out using SPSS (version 28.0.1.1). Among other things, the data was subjected to a one-way analysis of variance (ANOVA). Before this analysis was carried out, the normality of the residuals was tested using the Shapiro-Wilk test and the homogeneity of variance between harvest dates was tested using the Levene test. The significant differences between the harvest dates were calculated with the *post-hoc* analysis using the Tukey-test (p< 0.05). The standard deviation (SD) is marked in the corresponding tables as “±” and the significant differences with different lower-case letters.

### 2.6 Multivariate time-series classification and classification function derivation

The secondary metabolites content in tomatoes on the different harvest dates were grouped into predefined quality classes, 0 for low content and 1 for high content. The classification was based on the time-series containing the collected greenhouse data. There were in total N=8  time-series with M=6 measurement channels and with on average 57354 data points. Formally, given N time-series $X_{k}$ of different length $L_{k}$ with M channels, such that k=1,…,N and $X_{k}\in R^{L_{k}\times M}$ , the dataset $X=\left(X_{1},c_{1}\right),\ldots ,\left(X_{N},c_{N}\right)$ with $c_{k}\in 1,\ldots ,C$ where C is the number of classes (here C=1), the goal is to construct a classification function F(·) , such that $F\left(X_{test}\right)=c_{test}$ with $c_{test}\in 1,\ldots ,C$ and $X_{test}\in R^{L_{k}\times M}$ is the unseen time series, based on the training on the dataset X .

The classification function was derived as formally defined next. $X_{1}$ defines the subset of the given training X such that all $X_{k}\;\in$ $\;X_{1}$ have the class $c_{k}=1$ . The following quantities for the features of $X_{1}$ were considered, with

1. Minimum value $X_{1}^{min}$

2. Maximum value $X_{1}^{max}$

3. First quantile $X_{1}^{1st}$

4. Third quantile $X_{1}^{3rd}$

$X_{test}$ is the test time series with unknown class and $x^{\prime }:=mean(X_{test}$ ) the respective mean value of an univariate time series, i.e. mean values were computed for each channel separately as the time-series characteristics. Formally, let $X_{k}^{j}$ be the j -th channel of k -th time-series with the corresponding length $L_{k}$ . Then the standard formula reads as:


$$mean(X_{k}^{j}):=\;\frac{1}{L_{k}}\sum_{i=1}^{L_{k}}X_{k}^{j}\left(i\right)$$

The following functions were defined as the final classification function:


$$f_{c}\left(x,p_{1},p_{2}\right):=\;1,\;\;if\;p_{1}\leq x\leq p_{2}$$

0, otherwise

$$F\left(X_{test}\right):=max\left(f_{c}\left(x^{\prime },X_{1}^{min},X_{1}^{max}\right),\;f_{c}\left(x',X_{1}^{1st},X_{1}^{3rd}\right)\right)$$

where $p_{1},p_{2}$ are prescribed lower and upper bounds of the mean.

All of the aforementioned notions were implemented and calculated using R standard library (version 4.4.1). As an example consider the following small artificial training dataset $X:=\left\{\left(\left[1,2,3\right],0\right),\left(\left[2,3,4\right],1\right),\left(\left[2,3,5\right],1\right),\left(\left[3,4,6\right],1\right),\left(\left[7,8,9\right],0\right)\right\},\;X_{test}^{1}:=\left[6,7,8\right]$ and $X_{test}^{2}:=\left[3,4,5\right]$ . With mean as a chosen feature, $X_{1}=\{3,3.3333,4.3333)$, $X_{1}^{min}=3,\;X_{1}^{max}=4.3333,\;X_{1}^{1st}=3.167,\;\;X_{1}^{3rd}=\;3.833$ , $x^{1^{\prime }}=mean\left(X_{test}^{1}\right)=7$ and $x^{2^{\prime }}=mean\left(X_{test}^{2}\right)=4$ . The evaluation results in $F\left(X_{test}^{1}\right)\;=\;max\left(0,0\right)\;=\;0$ and $F\left(X_{test}^{2}\right)\;=\;max\left(1,0\right)\;=\;1$ . Thus, $X_{test}^{1}$ and $X_{test}^{2}$ receive class 0 and class 1 respectively.

The classes and class distributions were derived as follows: the determined contents of the secondary metabolites compounds can be seen as a collection of a 11-dimensional vector $\text{y}=\left(y_{c_{1}},y_{c_{2}},y_{c_{3}},y_{f_{1}},\ldots ,y_{f_{4}},y_{y_{ph_{1}}},\ldots ,{\text{y}}_{{\text{ph}}_{4}}\right)$ where $y_{c_{i}}$ correspond to the carotenoids contents and $y_{f_{j}},y_{ph_{j}}$ correspond to the flavonoid and phenolic compounds respectively. To explore the behavior of the compound vectors and to aggregate common characteristics, the tSNE-visualization (Van Der Maaten and Hinton, 2008) and k-means (MacQueen, 1967) algorithm using R (version 4.4.1) were applied. To ensure comparability across different compounds, the dataset was subjected to a normalization, which is crucial to adjust the range difference across the variables. Here, tSNE maps the higher dimensional data to the lower dimensional space and k-means clusters the resulting projected data points. Application of tSNE with the so-called perplexity parameter Perp=4 on all such compounds vectors results in a set of two-dimensional points, which allows to identify important patterns and clusters among the compounds. To identify distinct groups within the data, k-means clustering with k=2 was performed on the reduced-dimensionality data obtained from the t-SNE analysis. For each of the clusters the corresponding histogram for each compound component were constructed. These clusters were subsequently used to define two distinct classes within the compound dataset.

To validate and compare these classes, we constructed histograms to visualize the distribution of the corresponding secondary metabolite content values across each class. The objective of the visualization of the histograms is that one expects a clear separability of these data distributions between the classes and subsequentially the interpretability, i.e. one seeks a class combination, where it is clearly visible that for some compound one class characterizes low content and another separable class stands for the high content. After such compound is found, it is removed from the dataset and then the same procedure is applied again until all proper classes for all corresponding secondary metabolite compounds are determined.

### 2.7 Data augmentation

Because only N=8 time-series were available, which generally is a too small data set for the training of e.g. neural networks or support vector machines, the data augmentation technique was used to address this data limitation. Data augmentation techniques are used to generate synthetic time-series data that share some common patterns with the original dataset (Iglesias et al., 2023). Here the so-called moving block bootstrap method (Efron, 1979) was employed to generate 4000 new synthetic univariate time-series for each corresponding channel of the multivariate time-series of the original dataset. More specifically, to form new synthetic time series it was sampled with replacement l times from contiguous blocks of length b∈ℕ from the univariate time-series X of length $L_{k}$ . This produced $L_{k}\approx b\cdot l$ new synthetic time series, i.e. the moving block bootstrap sample $X^{\prime }$ . Thus, the new augmented dataset consisted of the training set itself and 500 new time series $X_{j}$ of length $L_{k}$ with b=650 and j=1,…,B , such that each $X_{j}$ has the corresponding class $c_{k}$ . For the bootstrapping purposes we utilized the R function tsbootstrap from the package tseries 0.10-56.

### 2.8 Accuracy calculation and cross validation

Our classification function was applied to the moving block bootstrapped augmented data to categorize the data into distinct classes according to the corresponding annotated secondary metabolite data. The performance of the classification model was evaluated using accuracy as the primary metric. An 8-fold cross validation (Kohavi, 1995) was performed on an moving-block bootstrapped augmented dataset, i.e. the training set consists of 7 original time series (augmented to 3500) and the goal is to predict the class of a single test time series (augmented to 500). This experiment is repeated for each of the 8 original time series.

During this classification process, each channel’s contribution to predicting the class of a particular secondary metabolite was evaluated. The channel that achieved the highest accuracy in classification was identified as having the highest impact. This analysis involved comparing the accuracy across different channels to determine which one provided the most reliable predictions.

The channel that achieved the highest accuracy in predicting the class of a particular secondary metabolite was identified as the factor with the highest impact. The optimal ranges for the sensor channels were determined through visual inspection of histograms depicting the mean values of the augmented time-series dataset. By examining the distribution of mean values across different classes, these specific ranges were identified that optimize the detection of classes with high secondary metabolites content.

**Figure 1:**
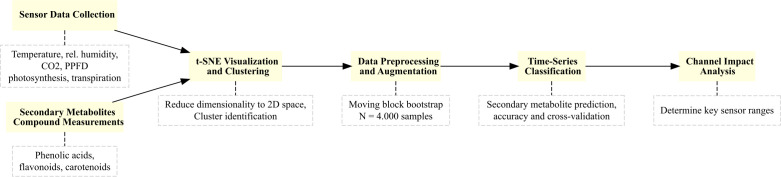
Workflow summarizing the methodology for secondary metabolite content prediction.

Finally, **Figure 1** illustrates workflow of the methodology we used to analyze and predict secondary metabolite content. The process begins with the collection of environmental and plant physiological data using sensors that measure variables such as temperature, relative humidity, CO_2_ concentration, PPFD, photosynthesis, and transpiration rates. Secondary metabolite compounds, including phenolic acids, flavonoids, and carotenoids, are subsequently quantified.

The raw data undergoes an augmentation by the application of a moving block bootstrap algorithm to generate synthetic datasets, which enhance model training. To reduce dimensionality and identify clusters, t-SNE visualization is employed. The workflow then proceeds to multivariate time-series classification to predict quality classes of metabolite content. Finally, channel impact analysis is performed to determine the most influential environmental factors, such as optimal PPFD and transpiration levels, that affect corresponding secondary metabolite accumulation.

## 3 Results and discussion

### 3.1 Abiotic factors, photosynthesis and transpiration during experiments

The following data show abiotic factors and their influence on plant physiological processes, specifically on photosynthesis and transpiration, over a period from June 11th to November 25th (**Table 1**). Generally, the seasonal variations in abiotic factors significantly impacted plant physiological processes. In detail, the average temperature ranged from 20.68 ± 3.06°C to 24.05 ± 4.24°C, with the highest average temperature recorded from June 11th to July 1st. Mean temperatures in this range generally enhance enzymatic activity, which can lead to increased photosynthetic rates (Sato et al., 2006; Dannehl et al., 2014a, b). The data show that the highest photosynthetic rate of 2.25 µmol CO2/m²s was observed from July 22nd to August 12nd, coinciding with a relatively high average temperature of 22.48°C. Conversely, the lowest photosynthetic rate of 0.17 µmol CO2/m²s was recorded from November 4th to November 25th, when the average temperature was 22.26°C, suggesting that other factors besides temperature may have influenced photosynthesis during this period. As such, CO2-concentration is a key determinant of photosynthetic rates. The CO2 concentrations varied between 530.79 ppm and 632.78 ppm, with the highest average concentration observed from November 4th to November 25th. Elevated CO2 levels can enhance photosynthetic rates by increasing the availability of CO2 for carbon fixation (Dannehl et al., 2021). However, the data show that the highest photosynthetic rate was not observed during the period with the highest CO2 concentration (**Table 1**), suggesting that another factor, such as light availability, may have played a more significant role in determining photosynthetic rates. In this context, the ambient photosynthetic photon flux density (PPFD) showed significant variability, with the highest average PPFD of 962.57 µmol/m²s from June 11th to July 1st and the lowest average PPFD of 178.19 µmol/m²s from November 4th to November 25th. Higher PPFD levels generally lead to increased photosynthetic rates, as light is the primary energy source for photosynthesis (Kläring and Krumbein, 2013). The data in the present study show that the highest photosynthetic rate was observed during the period with the highest PPFD, indicating a strong positive correlation between light availability and photosynthetic activity.

**Table 1:** Changing abiotic factors and plant responses during experiments.

Sampling	Photosynthesis [ µmol CO_2_/m^2^s]			Transpiration [mg H_2_O/m^2^s]			Temperature [°C]			Humidity [%]			CO_2_-concentration [ppm]			Ambient PPFD [µmol/m^2^s]		
June 11 – July 1	0.77	±	1.69	10.58	±	11.93	24.05	±	4.24	69.57	±	20.66	546.72	±	108.32	962.57	±	707.27
July 1 – July 22	0.73	±	1.58	5.66	±	8.27	20.68	±	3.06	78.03	±	20.08	553.04	±	94.44	659.15	±	578.91
July 22 – Aug 12	2.25	±	2.81	8.45	±	10.59	22.48	±	3.38	80.92	±	18.90	578.25	±	139.76	792.54	±	622.39
Aug 12 – Sept 2	1.06	±	1.73	6.48	±	7.61	21.04	±	3.76	79.43	±	18.85	587.79	±	125.70	775.14	±	587.90
Sept 2 – Sept 23	1.09	±	1.67	4.51	±	5.39	21.44	±	3.13	85.42	±	12.52	619.54	±	120.42	643.64	±	490.15
Sept 23 – Oct 14	0.81	±	1.45	3.77	±	3.08	21.52	±	2.33	88.95	±	8.23	576.14	±	146.19	377.89	±	353.42
Oct 14 – Nov 4	0.71	±	1.31	3.88	±	3.06	20.87	±	2.76	88.76	±	11.11	530.79	±	120.39	346.24	±	281.38
Nov 4 – Nov 25	0.17	±	0.76	3.50	±	2.13	22.26	±	2.03	79.50	±	9.34	632.78	±	85.56	178.19	±	177.47

The greenhouse data is given as mean value ± SD.

Furthermore, relative humidity is another important abiotic factor that affects plant water relations. The relative humidity ranged from 69.57% to 88.95%, with the highest average relative humidity recorded from September 23rd to October 14th. Higher relative humidity levels can reduce transpiration rates by decreasing the vapor pressure deficit, which is the driving force for water loss from plants (Sinclair et al., 2017). The data show that the highest transpiration rate of 10.58 H2O/m²s was observed from June 11th to July 1st, when the relative humidity was relatively low at 69.57%. Conversely, the lowest transpiration rate of 3.50 H2O/m²s was recorded from November 4th to November 25th, when the relative humidity was 79.50%. This inverse relationship between humidity and transpiration is consistent with the expected physiological response of plants to changes in atmospheric moisture.

In summary, our data reveal complex interactions between abiotic factors and plant physiological processes. Temperature, relative humidity, CO2-concentration, and PPFD all play crucial roles in determining photosynthetic and transpiration rates. Higher temperatures and PPFD levels generally enhanced photosynthesis, while higher relative humidity levels reduced transpiration rates. However, the data also suggest that the combined effects of these factors can lead to non-linear responses in plant physiological processes. For example, the highest photosynthetic rate was not observed during the period with the highest CO2 concentration, indicating that other factors, such as temperature and light availability, may have been more influential.

### 3.2 Seasonal behavior of secondary plant compounds

Generally, the contents of carotenoids in tomatoes are consistent with those detected in other studies (Frusciante et al., 2007; Dannehl et al., 2012; Flores et al., 2017). The analysis revealed that lycopene was the predominant carotenoid, with ß-carotene and lutein being the subsequent most prevalent (**Table 2**). Our data indicate complex seasonal fluctuations in the carotenoid composition of tomatoes throughout the harvest year (**Table 2**), which were also found in other studies (Dumas et al., 2003; Raffo et al., 2006). The peak concentrations of all carotenoids were predominantly observed in August and September, with lower levels detected at earlier and later harvest periods in comparison. In detail, the lutein content had a clear peak value in mid-August. This value of 15.12 µg/g DW is not only highly significantly different from all other measured values, but also more than twice as high as the values in October and early November and up to three times as high as the values in early summer and those in September and late November.

**Table 2:** Changes in carotenoids during the growing season.

Sampling	Lutein				ß-carotene				Lycopene				Total carotenoids			
June 11	5.70	±	0.66	b	35.23	±	1.81	c	470.47	±	94.56	bcd	511.40	±	95.08	bcd
July 1	4.10	±	0.56	b	16.71	±	0.84	d	202.68	±	19.98	e	223.49	±	18.63	d
July 22	5.60	±	3.17	b	69.08	±	13.21	ab	720.47	±	31.58	abc	795.16	±	21.26	abc
Aug 12	15.12	±	1.69	a	90.41	±	27.22	a	878.32	±	321.09	ab	983.85	±	348.92	ab
Sept 2	4.81	±	0.94	b	74.19	±	11.77	ab	1148.82	±	264.88	a	1227.82	±	275.18	a
Sept 23	4.72	±	0.30	b	74.63	±	4.64	ab	1122.09	±	161.00	a	1201.44	±	164.48	a
Oct 14	7.23	±	0.24	b	48.59	±	4.82	bc	644.70	±	83.42	bcd	700.52	±	87.91	bcd
Nov 4	7.23	±	1.30	b	47.41	±	6.07	bc	394.72	±	124.51	cd	449.36	±	131.34	cd
Nov 25	4.00	±	0.42	b	31.07	±	5.83	c	499.76	±	57.99	bcd	534.83	±	64.24	bcd

The content of carotenoids is given as mean value in µg/g DW ± SD. Significant differences are shown as different letters and calculated using Tukey`s HSD-Test at a significance level of p< 0.05 (n = 3).

The content of ß-carotene, on the other hand, rose continuously and significantly from an initial level of 35.23 µg/g DW in June to a maximum value of 90.41 µg/g DW in August except for early July, remained there in September at approximately 75 µg/g DW and then fell continuously until the end of November (31.07 µg/g DW), significantly lower than the August value.

Almost the same pattern was also found in terms of the lycopene accumulation. Lycopene followed a positive trend over the summer with a clear high plateau in September (maximum 1148.82 µg/g DW), whose values differed highly significantly from both those from early summer (minimum 202.68 µg/g DW) and late fall (minimum 394.72 µg/g DW).

Finally, the total carotenoid levels also confirmed this seasonal trend. They rose significantly up to the same plateau in September, where they reached their highest level (1227.82 µg/g DW). This was more than twice as high as the starting point in June (511.40 µg/g DW).

This indicates that the increase in carotenoids is a temporary phenomenon that is influenced by specific environmental factors, such as temperature and PPFD as shown by Slimestad and Verheul (2005) and Formisano et al. (2021). Both studies were able to demonstrate correlations between these abiotic factors and the accumulation of carotenoids, but did not indicate a specific range in which the carotenoids accumulate the most. A multivariate analysis was also not shown and will be considered in the present study as shown below.

Phenolic acids were found to dominate, accounting for 83% of detected total phenolic compounds, followed by flavonoids at 17%. These results confirm those reported by Cruz-Carrión et al. (2022). In particular, caffeic acid derivatives were the ones with the maximum occurring content (567.24 µg/g DW), followed by coumaric acid hexoside (469.22 µg/g DW), caffeoylquinic acid derivatives (413.70 µg/g DW), ferulic acid hexoside (101.07 µg/g DW) and coumaroylquinic acids (95.00 µg/g DW) (**Table 3**). Under consideration of phenolic acids in tomatoes, the contents of all phenolic acids varied between the harvest dates, although the fruits were harvested at the same ripening stage (**Table 3**). The same was found under conditions in New Zealand (Toor et al., 2006). As observed by Raffo et al. (2006) we were also able to demonstrate that the accumulation of a variety of phenolic compounds decreases in the warm season and increases in the cooler season. In detail, the initial value of the coumaric acid hexoside content in June (180.94 µg/g DW) was significantly lower than the subsequent measured values, except for the measured content at the beginning of July. This was followed by a gradual increase until August with a significantly higher value of 329.55 µg/g DW. In September, the content dropped slightly (265.71 µg/g DW), but then rose again to its significantly highest value at the beginning of November (469.22 µg/g DW). Similar was found for ferulic acid hexoside. Prior to September, the content of ferulic acid hexoside increased gradually from 46.41 µg/g DW on June 11th to 49.40 µg/g DW on July 22nd, with no significant differences between the individual harvest times. However, a marked increase in ferulic acid hexoside content occurred after September, with values rising to 93.17 µg/g DW in October and peaking at 101.07 µg/g DW on November 4.

**Table 3:** Seasonal changes in phenolic acids in tomatoes.

Sampling	Coumaric acid hexoside				Ferulic acid hexoside				Caffeic acid derivates sum				Caffeoylquinic acids derivates sum				Coumaroylquinic acids sum				Total phenolic acids			
June 11	180.94	±	12.63	f	46.41	±	3.97	a	211.16	±	5.44	d	170.76	±	19.65	c	95.00	±	5.64	a	704.26	±	35.74	de
July 1	102.85	±	10.66	g	48.86	±	2.62	a	189.45	±	5.39	d	218.41	±	13.27	bc	45.53	±	9.06	b	605.09	±	41.01	e
July 22	218.07	±	6.17	ef	49.40	±	5.07	a	285.54	±	10.94	c	244.05	±	35.96	bc	27.69	±	2.80	b	824.76	±	52.79	de
Aug 12	329.55	±	26.85	cd	70.33	±	4.25	b	567.24	±	56.56	a	405.98	±	46.23	a	44.56	±	27.38	b	1417.67	±	90.26	ab
Sept 2	265.71	±	23.99	de	74.19	±	2.22	b	491.20	±	18.78	a	252.88	±	86.99	bc	38.47	±	7.73	b	1122.46	±	73.41	c
Sept 23	346.02	±	41.23	bc	86.35	±	4.95	ab	531.54	±	43.35	a	200.06	±	21.63	c	44.75	±	8.57	b	1208.72	±	109.94	bc
Oct 14	421.61	±	25.96	ab	93.17	±	9.28	a	495.84	±	30.12	a	334.48	±	56.65	ab	57.08	±	8.97	b	1402.18	±	115.05	ab
Nov 4	469.22	±	33.15	a	101.07	±	9.89	a	524.57	±	15.90	a	413.70	±	34.92	a	40.36	±	4.71	b	1569.22	±	76.88	a
Nov 25	265.62	±	42.48	de	75.07	±	5.54	b	376.93	±	4.39	b	140.99	±	34.42	c	4.21	±	0.85	c	862.81	±	63.20	d

The content of flavonoids is given as mean value in µg/g DW ± SD. Significant differences are shown as different letters and calculated using Tukey`s HSD-Test at a significance level of p< 0.05 (n = 3).

Further results showed a significant increase in caffeic acid derivatives over time, with distinct changes in the trend around August and September. Prior to August, the content of caffeic acid derivatives decreased from 211.16 µg/g DW on June 11th to 189.45 µg/g DW on July 1st, and then significantly increased to 285.54 µg/g DW on July 22nd. From July until November 4th, a consistent and significant increase in caffeic acid derivatives was observed, with values ranging from 491.20 µg/g DW to 567.24 µg/g DW. However, a significant decline in caffeic acid derivatives content was observed on November 25th (376.93 µg/g DW).

Furthermore, our results demonstrate a clear trend of increasing caffeoylquinic acids derivatives content from early to late harvest periods, with a peak in August and November, followed by a decline towards the end of the season. The results show that the highest content of caffeoylquinic acids derivatives was found in tomatoes harvested on August 12th (405.98 µg/g DW) and November 4th (413.70 µg/g DW), which were not significantly different from each other. Tomatoes harvested on November 4th had the lowest content of caffeoylquinic acids derivatives (140.99 µg/g DW). The intermediate levels of caffeoylquinic acids derivatives ranged between 170.76 and 252.88 µg/g DW mostly found during the first three harvest dates and in September, with no significant differences among these dates.

The analysis of the contents of coumaroylquinic acids in tomatoes revealed a significant change over the harvest time. The highest values were observed on June 11th (95.00 µg/g DW). In summer and autumn, the values decrease significantly, but, except for July 2nd, remained relatively constant between 38.47 and 57.08 µg/g DW), before rapidly decreasing to its lowest value at the end of November (4.21 µg/g DW).

Based on the behavior of the individual phenolic acid, the initial three harvest dates exhibited relatively low levels of total phenolic acids, ranging from 605.09 to 824.76 µg/g DW, with no significant variation among them. A sharp and significant increase was observed in August, peaking at 1417.67 µg/g DW, followed by a brief decline in early September to 1122.46 µg/g DW. Subsequently, the total phenolic acids steadily increased until November 4th reaching its maximum value of 1569.22 µg/g DW; a level that is two and a half times higher than the lowest value detected on July 1st. At the end of the experiments, the total phenolic content decreased significantly by half compared to this peak value.

Based on the flavonoids we detected and regardless of the time of harvest, quercetin derivatives were the most abundant flavonoids (200.50 µg/g DW), followed by naringenin (61.06 µg/g DW) and phloretin diglucodise (46.67 µg/g DW) (**Table 4**). The same was reported by Abreu et al. (2019). Our data reveal that the content of naringenin in tomatoes exhibits variability over the harvest time points. The highest values were recorded in June 11th (61.06 µg/g DW) and November 4th (47.23 µg/g DW), with a slight increase towards the end of the season. The lowest values occurred in mid-August (15.32 µg/g DW), with values preceding and following this period remaining at a similar level.

**Table 4:** Seasonal effects on the flavonoid content of tomatoes.

Sampling	Naringenin				Quercetin				Phloretin diglucoside				Total flavonoids			
June 11	61.06	±	10.81	a	92.38	±	4.64	c	33.27	±	1.55	abc	186.70	±	12.01	abc
July 1	39.37	±	5.67	abc	107.70	±	0.22	c	26.38	±	1.81	bcd	173.45	±	7.70	c
July 22	42.86	±	16.60	abc	119.99	±	20.13	bc	18.23	±	3.76	c	181.08	±	27.07	bc
Aug 12	15.32	±	0.71	c	200.50	±	29.10	a	33.88	±	6.89	abc	277.18	±	58.93	a
Sept 2	30.16	±	10.10	bc	150.88	±	49.76	abc	30.17	±	5.03	bcd	202.66	±	70.67	abc
Sept 23	22.49	±	13.35	bc	130.74	±	14.03	bc	46.65	±	4.25	a	199.89	±	18.61	abc
Oct 14	46.07	±	6.25	ab	147.97	±	9.45	abc	42.58	±	1.48	ab	236.62	±	2.81	abc
Nov 4	47.23	±	6.75	ab	181.88	±	10.14	ab	42.96	±	5.46	ab	272.07	±	15.17	ab
Nov 25	42.62	±	12.73	abc	137.49	±	4.39	bc	46.67	±	8.97	a	226.78	±	12.27	abc

The content of flavonoids is given as mean value in µg/g DW ± SD. Significant differences are shown as different letters and calculated using Tukey`s HSD-Test at a significance level of p< 0.05 (n = 3).

Furthermore, the data demonstrate a significant increase in the content of quercetin in tomatoes, rising from 92.38 μg/g DW in June 1 to a peak of 200.50 μg/g in August 12th. Subsequently, the levels of quercetin remain at a similarly high level (130.47 to 181.88 μg/g DW), which is distinctly higher than that observed in earlier-collected samples (92.38 to 119.99 μg/g DW).

The analysis of further data reveals a significantly low content of phloretin diglucoside in tomatoes on July 22nd (18.23 μg/g DW), in contrast to a peak value of 46.67 μg/g DW on November 25. Upon closer examination, the contents can be divided into two seasonal groups, with lower levels observed during summer months and higher levels during autumn months, which are relatively constant (42.58-46.67 μg/g DW) compared to those detected during summer months (18.23-33.88 μg/g DW).

Based on the data, the highest total flavonoid content was achieved on August 12th in midsummer (277.18 µg/g DW), which was significantly different from the contents determined in the previous month. All other determined total flavonoid contents were not significantly different from this value. However, when looking at the overall trend, it is evident that the flavonoid contents of tomatoes ripened in June and July were lower (173.45 to 186.70 µg/g DW) compared to those accumulated in the fruits from August to November (199.89 to 277.18 µg/g DW).

All the mentioned results impressively underline the major role played by the time of harvest when it comes to achieving maximum nutrient values regarding the secondary plant compounds in tomatoes. In this context, the accumulation of secondary metabolites in the seasonal course is thus characterized by the influence of various abiotic factors such as temperature (Krumbein et al., 2012), light intensity (Thoma et al., 2020) and CO2-concentration (Boufeldja et al., 2023), which has already been shown in previous studies on the basis of individual correlations. However, it can be assumed that individual abiotic factors are not sufficient to optimize the accumulation of secondary metabolites, as the climate is always composed of several abiotic factors. In addition, it is likely that the accumulation of secondary metabolites could also be explained by plant responses such as photosynthesis and transpiration. Therefore, all abiotic climate variables and plant responses will be considered as variables in the following calculations in order to make statements on the optimal conditions for the synthesis of secondary metabolites. To achieve this, a classification approach will be employed, where temperature, radiation, relative humidity, CO2 levels, photosynthesis and transpiration rates are used as input to predict the individual and total secondary metabolite content class as either high or low. On the one hand, classification will help to identify which conditions are most likely to result in high metabolite production. On the other hand, it simplifies complex, continuous greenhouse data into two discrete categories, making it easier for the user to interpret. Furthermore, the classification procedure will predict the class of metabolite content for new, unseen data, aiding in plant production planning and optimization.

### 3.3 Annotation of secondary plant compound measurements

Annotation of the dataset is a crucial step in preparation of the data for the classification. Being a supervised learning method, classification requires labelled data in order to train the resulting predictive model. Descriptive statistics was applied to annotate the secondary plant compound measurements in a low content (class 0) and high content (class 1). The following classes were obtained using repeated application of tSNE, k-means and respective histogram comparisons.

**Table 5** presents resulting statistics for carotenoid levels, including ß-carotene, lutein, lycopene, and total carotenoids, across two classes: Class 0 (low) and Class 1 (high). For ß-carotene, the maximum value in Class 0 is 50.97, while in Class 1, the minimum is 51.76, indicating a significant difference between the two classes. The mean values also show a clear distinction, with Class 0 at 34.66 and Class 1 at 75.13. Lutein and lycopene follow a similar pattern, with higher values in Class 1 across all statistics. Total carotenoids show the most significant overlap, with Class 0’s maximum (633.62) being lower than Class 1’s minimum (715.42), highlighting a clear separation between the two quality classes. The mean values for total carotenoids are 443.81 for Class 0 and 1010.85 for Class 1, further highlighting the difference.

**Table 5:** Descriptive statistics and annotation of carotenoids.

Statistics	ß-carotene		Lutein		Lycopene		Total carotenoids	
	Class 0 (low)	Class 31 (high)	Class 30 (low)	Class 31 (high)	Class 30 (low)	Class 31 (high)	Class 30 (low)	Class 31 (high)
Min	16.13	51.76	2.05	3.76	180.50	659.90	198.68	715.42
1st Qu.	26.64	68.83	3.79	5.08	264.30	717.40	294.73	791.31
Median	35.50	73.17	4.51	6.49	435.20	826.00	475.21	905.66
Mean	34.66	75.13	4.85	7.82	404.30	927.90	443.81	1010.85
3rd Qu.	42.39	79.77	5.93	7.94	516.70	1137.70	565.02	1225.41
Max	50.97	121.3	8.15	17.08	574.50	1426.90	633.62	1565.28

Content is given in µg/gDW.

As of flavonoids (**Table 6**), for naringenin, the mean values are 32.64 for Class 0 and 45.75 for Class 1, however, with a noticeable overlap in their ranges. Quercetin shows a mean of 111.80 for Class 0 and 166.70 for Class 1, with less overlap, especially at higher values. Phloretin diglucoside has means of 27.73 and 44.35 for Class 0 and Class 1, respectively, with some overlap. Total flavonoids have means of 443.81 for Class 0 and 1010.85 for Class 1, with a significant range difference.

**Table 6:** Descriptive statistics and annotation of flavonoids.

Statistics	Naringenin		Quercetin		Phloretin diglucoside		Total flavonoids	
	Class 0 (low)	Class 1 (high)	Class 0 (low)	Class 1 (high)	Class 0 (low)	Class 1 (high)	Class 0 (low)	Class 1 (high)
Min	4.525	26.54	87.88	105.40	14.99	36.70	198.68	715.42
1st Qu.	15.88	41.28	101.13	141.60	24.56	41.06	294.73	791.31
Median	36.70	47.50	107.57	153.20	28.47	43.78	475.21	905.66
Mean	32.64	45.75	111.80	166.70	27.73	44.35	443.81	1010.85
3rd Qu.	42.10	53.14	123.07	187.10	33.88	46.79	565.02	1225.41
Max	72.80	59.73	142.47	283.00	35.33	56.73	633.62	1565.28

Content is given in µg/gDW.

In terms of phenolic acids in **Table 7**, coumaric acid hexoside, Class 1 consistently shows higher values across all statistical measures compared to Class 0, with a mean of 437.70 in Class 1 against 236.74 in Class 0. Similarly, ferulic acid hexoside and caffeic acid derivatives also exhibit higher values in Class 1, with means of 83.37 and 522.10, respectively, compared to 47.96 and 265.80 in Class 0. The data for caffeoylquinic acids derivatives and coumaroylquinic acids also follow this trend, with Class 1 showing higher values. Notably, the total phenolic acids sum shows a significant difference, with Class 1 having a mean of 1010.85 compared to 443.81 in Class 0.

**Table 7:** Descriptive statistics and annotation of flavonoids.

Statistics	Coumaric acid hexoside		Ferulic acid hexoside		Caffeic acid derivates sum		Caffeoylquinic acids derivates sum		Coumaroylquinic acids sum		Total phenolic acids	
	Class 0 (low)	Class 1 (high)	Class 0 (low)	Class 1 (high)	Class 0 (low)	Class 1 (high)	Class 0 (low)	Class 1 (high)	Class 0 (low)	Class 1 (high)	Class 0 (low)	Class 1 (high)
Min	90.54	391.50	42.39	66.85	183.20	462.00	108.30	274.10	3.23	21.54	198.68	715.42
1st Qu.	190.15	410.80	45.51	72.88	203.50	500.30	173.90	327.30	9.71	36.81	294.73	791.31
Median	239.21	443.30	47.79	81.21	246.60	506.80	194.20	389.00	29.25	49.07	475.21	905.66
Mean	236.74	437.70	47.96	83.37	265.80	522.10	191.30	376.70	26.02	55.12	443.81	1010.85
3rd Qu.	310.71	450.10	50.33	90.58	316.00	533.50	223.00	430.50	35.02	69.28	565.02	1225.41
Max	360.26	507.40	54.63	108.13	380.20	609.70	253.90	474.60	51.56	99.08	633.62	1565.28

Content is given in µg/gDW.

There is a clear overlap in the ranges of values between the two classes, but the higher quantiles and maximum values in Class 1 indicate a distinct separation in phenolic acids content. The minimum values also highlight this difference, with Class 1 starting at much higher levels than Class 0.

### 3.4 Class assignments for the time-series measurements of greenhouse parameters

The class assignments of the corresponding time-series with respect to the individual and total secondary metabolites are illustrated in **Table 8**. This table provides an overview of how each time-series segment is categorized in relation to the corresponding metabolite content class. That is, strictly speaking, each row defines a separate classification task. Specifically, during the initial sampling period from June 11th to July 1st, all carotenoids start in class 0. This changes in the subsequent period from July 1st to July 22nd, where all carotenoids are in class 1, reflecting a higher carotenoid content. This high content continues consistently through the periods of July 22nd to August 12th, August 12th to September 2nd, and September 2nd to September 23rd. Starting from September 23rd to October 14th, there is a decline, with all carotenoids reverting to class 0. This lower assignment persists through the subsequent periods of October 14th to November 4th and November 4th to November 25th.

**Table 8:** Class assignments with respect to the individual and total secondary metabolites.

	Sampling							
	June 11 – July1	July1 – July 22	July 22 – Aug 12	Aug 12 – Sept 2	Sept 2 – Sept 23	Sept 23 – Oct 14	Oct 14 – Nov 4	Nov 4 – Nov 25
Carotenoids								
lutein	0	1	1	1	1	0	0	0
ß-carotene	0	1	1	1	1	0	0	0
lycopene	0	1	1	1	1	0	0	0
total carotenoids	0	1	1	1	1	0	0	0
Phenolic acids								
coumaric acid hexoside	0	0	0	1	1	1	1	0
ferulic acid hexoside	0	0	0	1	1	1	1	0
caffeic acid derivates sum	0	0	0	1	1	1	1	0
caffeoylquinic acids derivates sum	0	0	1	0	0	1	1	0
coumaroylquinic acids sum	1	0	0	1	1	1	0	0
total phenolic acids	0	0	1	0	1	1	1	0
Flavonoids								
naringenin	0	1	0	1	1	0	0	0
quercetin	0	0	0	1	1	1	1	0
phloretin diglucoside	0	0	0	0	1	1	1	1
total flavonoids	0	0	1	0	0	1	1	1

Class 0 encodes low content, while Class 1 encodes high content.

During the initial period from June 11th to July 1st, all flavonoids are assigned as class 0. In the subsequent period from July 1st to July 22nd, naringin improves class 1st, while the others remain at 0. From July 22nd to August 12th, the total flavonoids reach class 1, indicating an overall improvement despite individual components remaining at 0. The period from August 12th to September 2nd sees both naringin and quercetin reaching class 1, while phloretin diglucoside remains at 0, showing a partial improvement in flavonoid quality. From September 2nd to September 23rd, all flavonoids reach class 1, marking a peak in individual flavonoid quality. Furthermore, from September 23rd to October 14th, quercetin, phloretin diglucoside, and total flavonoids are all at class 1, indicating a shift in content dynamics. This pattern continues from October 14th to November 4th, with the same flavonoids maintaining high content, while naringin drop to 0. Finally, from November 4th to November 25th, only phloretin diglucoside and total flavonoids remain at class 1.

During the initial period from June 11th to July 1st, most phenolic acids are classified as 0, except for coumaroylquinic acids, which are in class 1. In the following period from July 1st to July 22nd, all phenolic acids drop to class 0. From July 22nd to August 12th, caffeoylquinic acid derivatives improve to class 1, while others remain at 0. The period from August 12th to September 2nd marks an improvement, with coumaric acid hexoside, ferulic acid hexoside, and caffeic acid derivatives all reaching class 1, although total phenolic acids remain at 0. From September 2nd to September 23rd, the quality of these acids remains high, and total phenolic acids also reach class 1, indicating a peak in overall content. This high content is sustained from September 23rd to October 14th, with all phenolic acids in class 1, marking the period of optimal quality. From October 14th to November 4th, most phenolic acids maintain their high content, except for coumaroylquinic acids, which drop to class 0, yet total phenolic acids remain at class 1. Finally, from November 4th to November 25th, all phenolic acids revert to class 0.

Often, classification cannot be directly performed on the raw time series data as it requires certain feature selection strategies (Liu, 2010). Specifically, we selected the mean of the time-series data as the sole feature for classification. This approach proved itself very effective, since, if successful, the mean provides a direct relationship to the desired secondary metabolite content. Using our classification function and cross validation, the resulting accuracies were computed. The mean accuracy of the cross-validation procedure provides the metric on how well the classification predicts the unseen data. Furthermore, the following hypothesis has been made - the higher the accuracy, the more impact has the channel to the accumulation of the corresponding metabolite content. Furthermore, as one can see from **Table 8**, there are only eight sampling time points, where the measurements of the secondary metabolites content took place. Due to this limitation, data augmentation using moving block bootstrap was applied to enhance the size of the dataset. Essentially, data augmentation ensures that our classification function is trained on a richer dataset, ultimately leading to a more accurate outcome.

For the main result, the histograms of the mean values of augmented time series alongside with the corresponding labelling of the secondary metabolite content are presented in **Table 9** for individual secondary metabolites and **Table 10** for the total secondary metabolites. Finally, the necessary ranges for the highest content were given by the visual inspection of the non-overlapping segments of Class 1 (high secondary metabolite content).

**Table 9:** Greenhouse parameter channels against individual secondary metabolites.

Channel	Group 1	Group 2	Naringenin	Phloretin diglucoside	Coumaroylquinic acids sum	Caffeoylquinic acids derivatives sum
**Temperature** **[°C]** **x=[18, 28]** **y=[0, 500]**	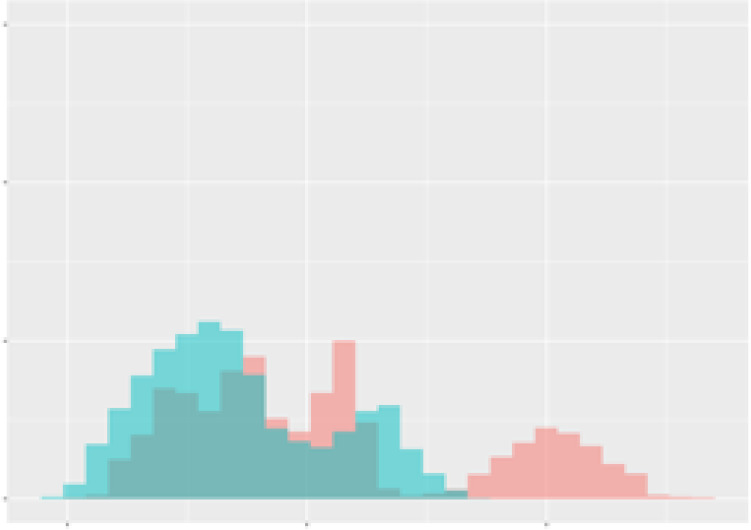 72.7 ± 13.47 [%]	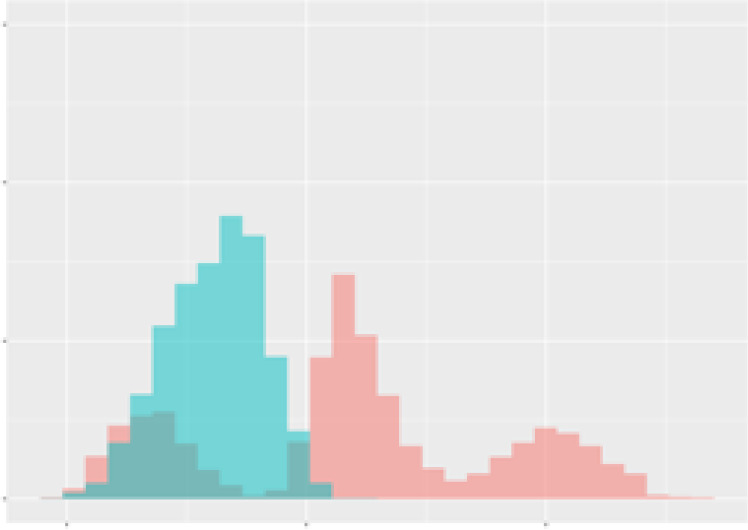 97.20 ± 2.66 [%] *)	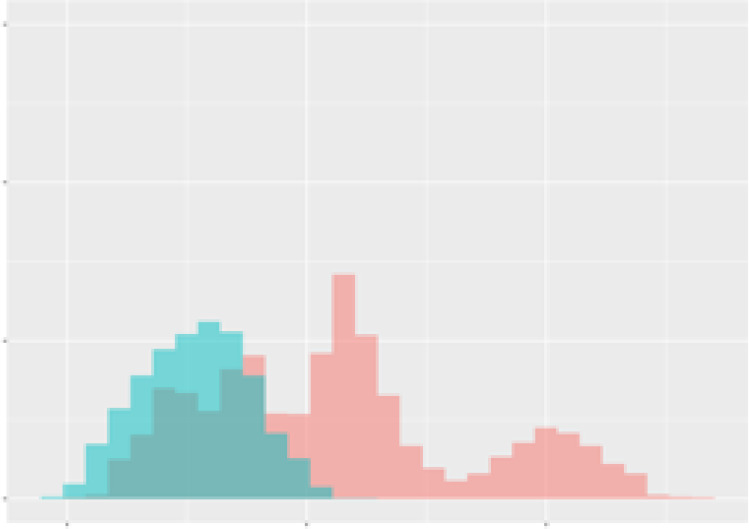 88.1 ± 8.72 [%]	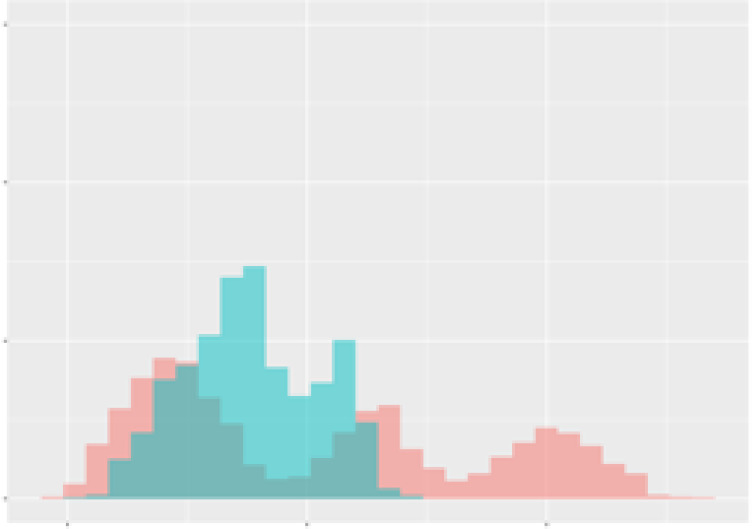 87.08 ± 5.94 [%]	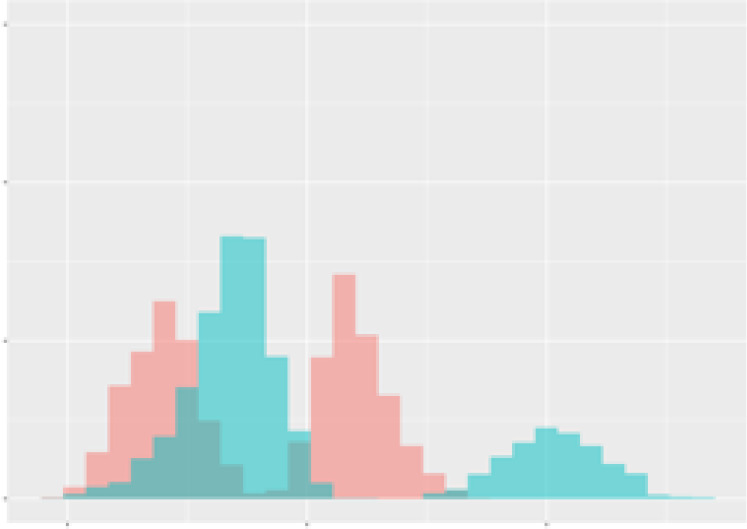 65.98 ± 15.44 [%]	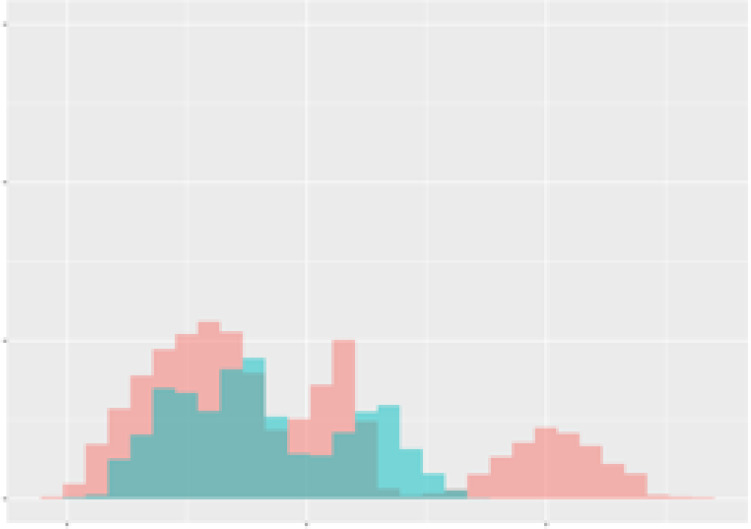 53 ± 13.25 [%]
**Photosynthesis** **[µmol CO2/qm s]** **x=[-16, 1.5]** **y=[0,1200]**	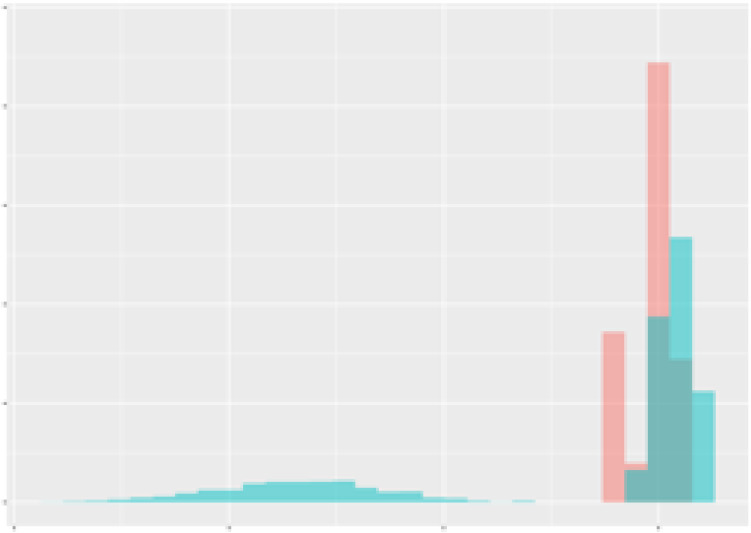 37.35 ± 18.09 [%]	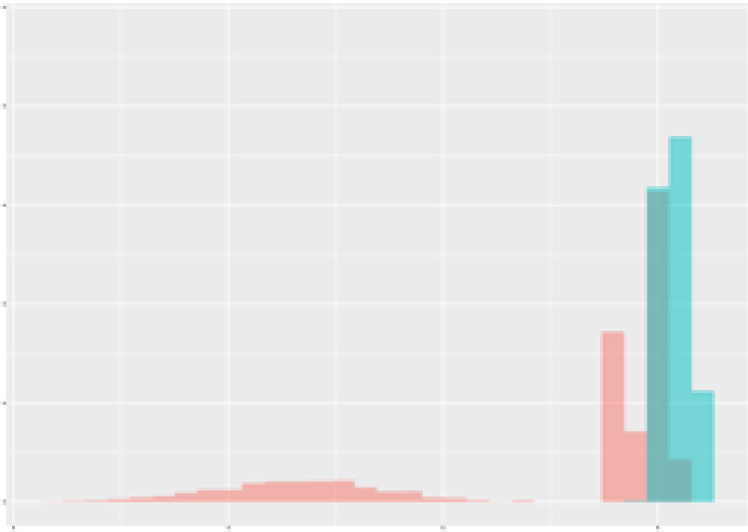 93.33 ± 3.90 [%] *)	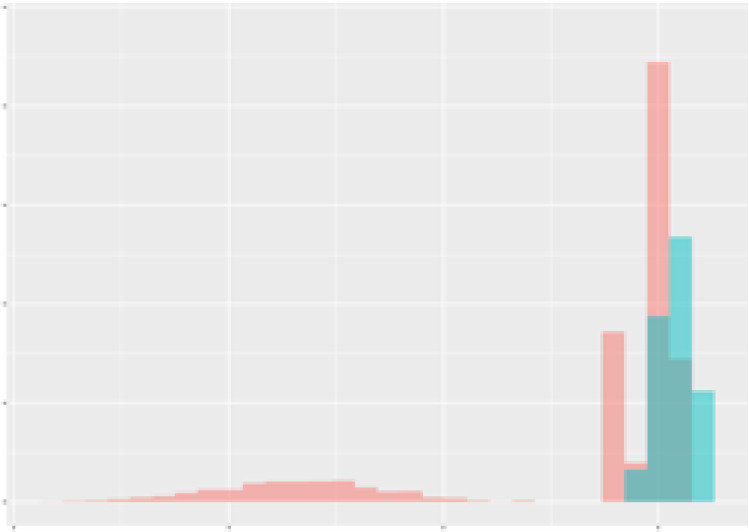 64.58 ± 13.77 [%]	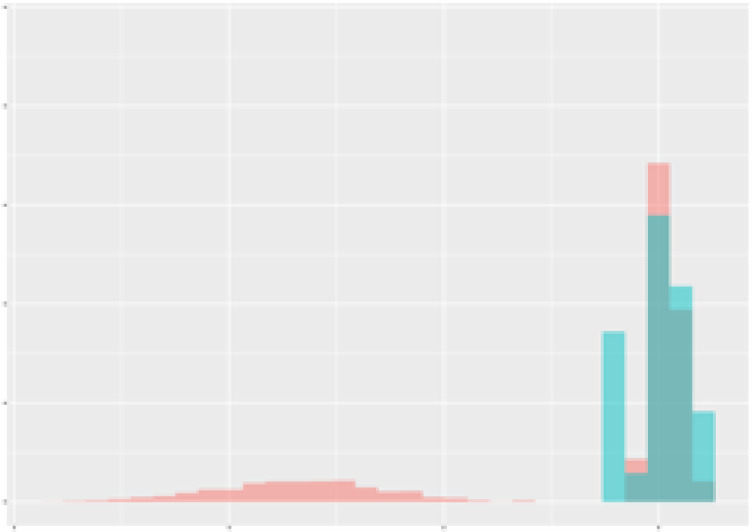 51.18 ± 15.94 [%]	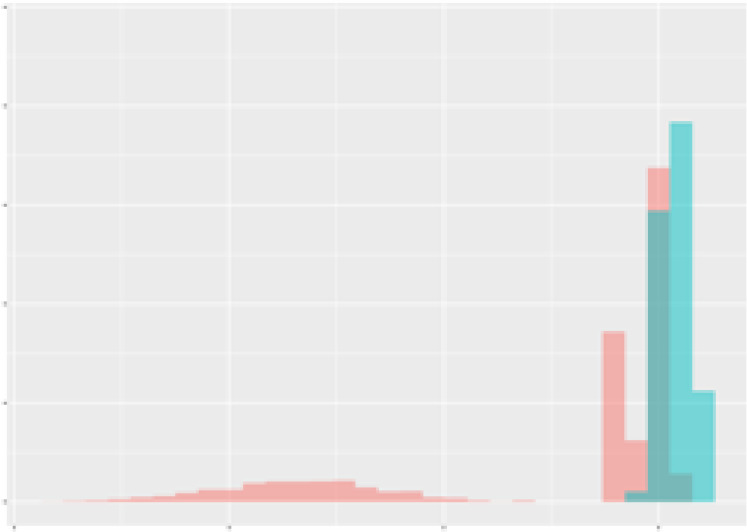 92.43 ± 3.88 [%] *)	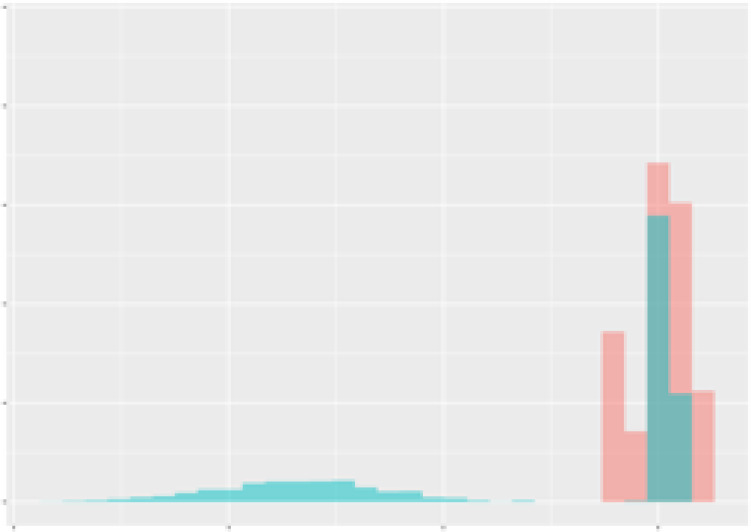 51.83 ± 17.20 [%]
**Transpiration** **[mg H2O/qm s]** **x=[3, 15]** **y=[0,750]**	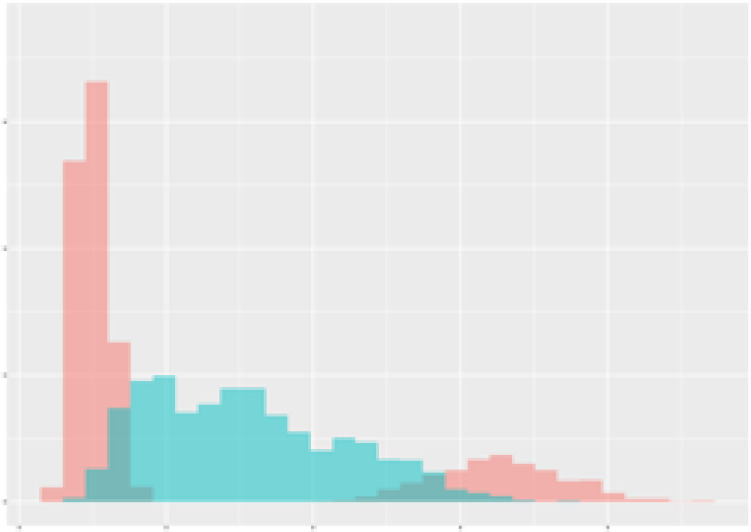 96.75 ± 3.03 [%] *)	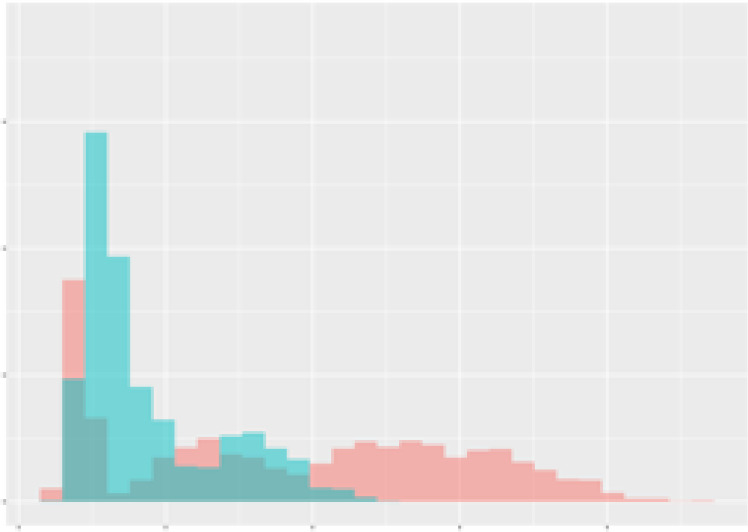 85.425 ± 10.28 [%]	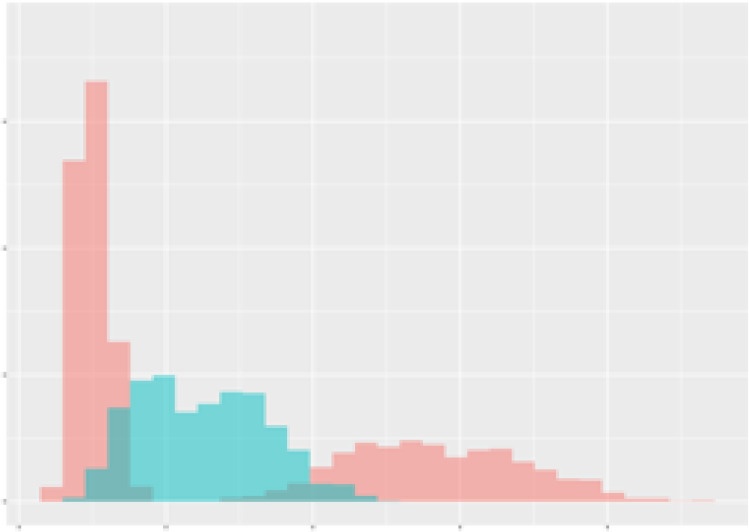 99.63 ± 0.20 [%] *)	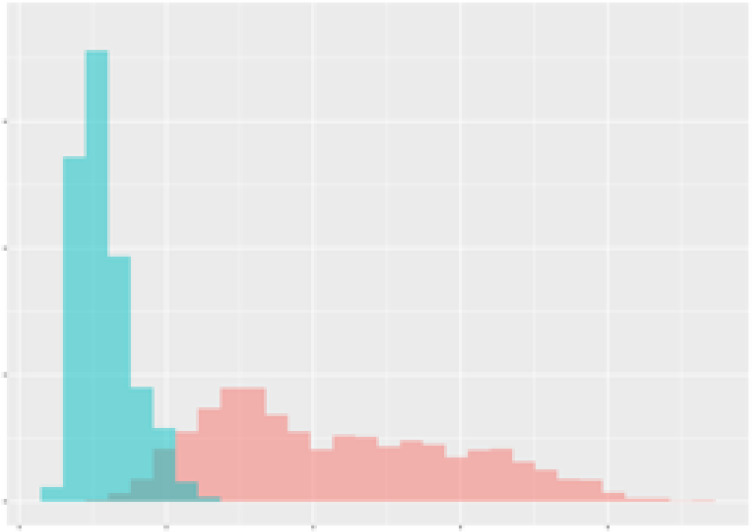 94.78 ± 4.94 [%] *)	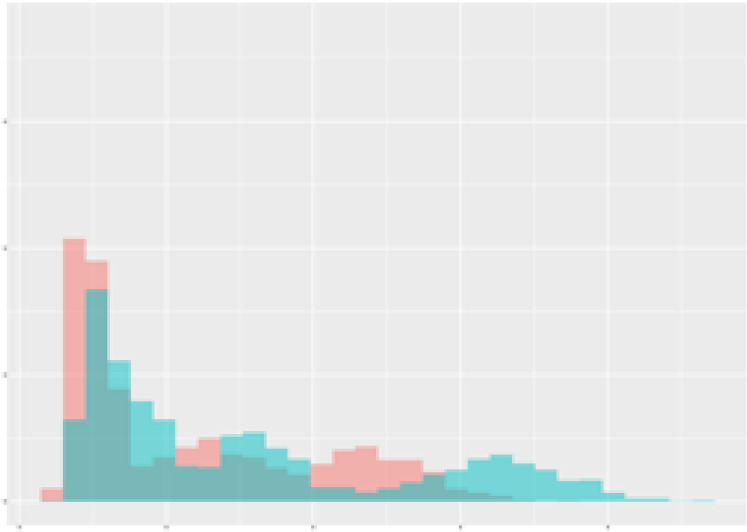 68.58 ± 14.66 [%]	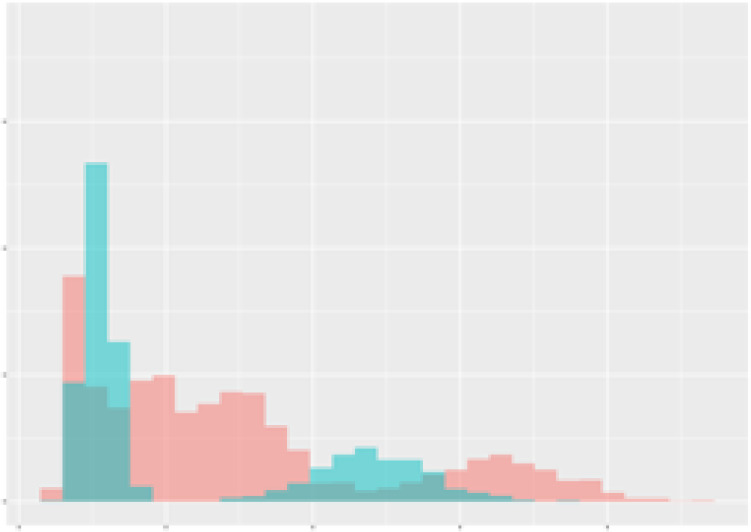 49.775 ± 18.1 [%]
**Humidity** **[%]** **x=[60, 95]** **y=[0,500]**	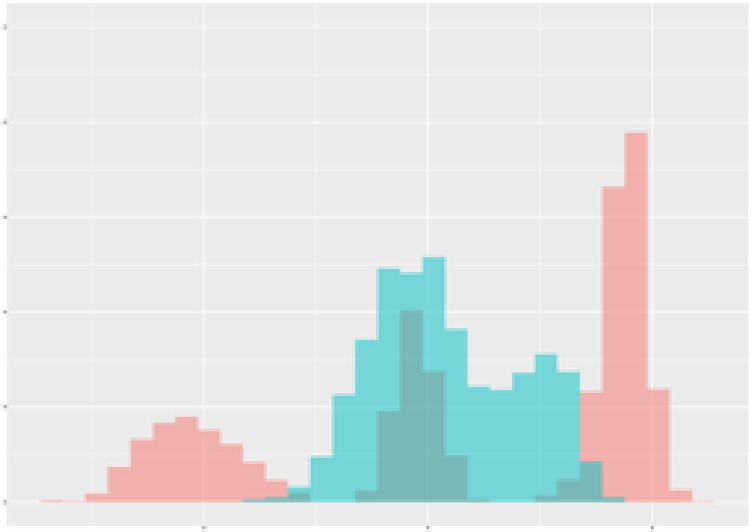 85.00 ± 11.41 [%]	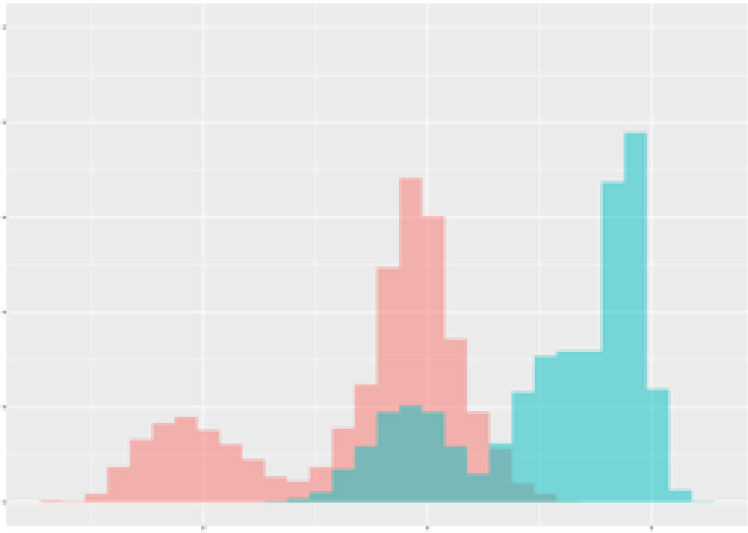 87.43 ± 11.21 [%]	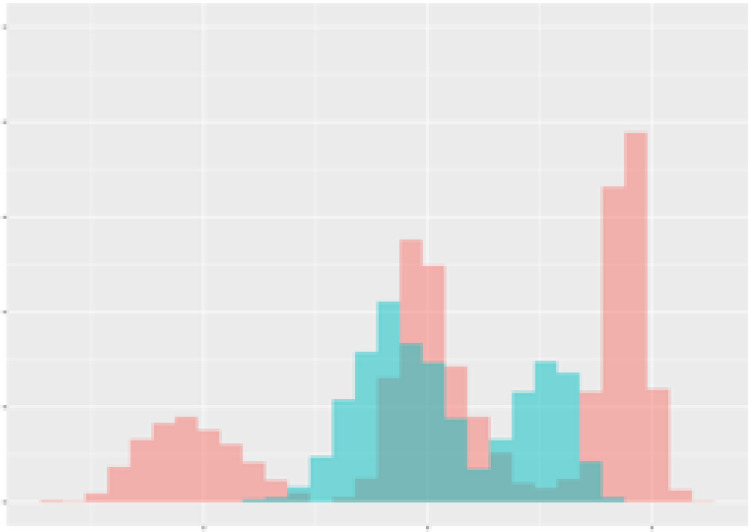 68.60 ± 15.74 [%]	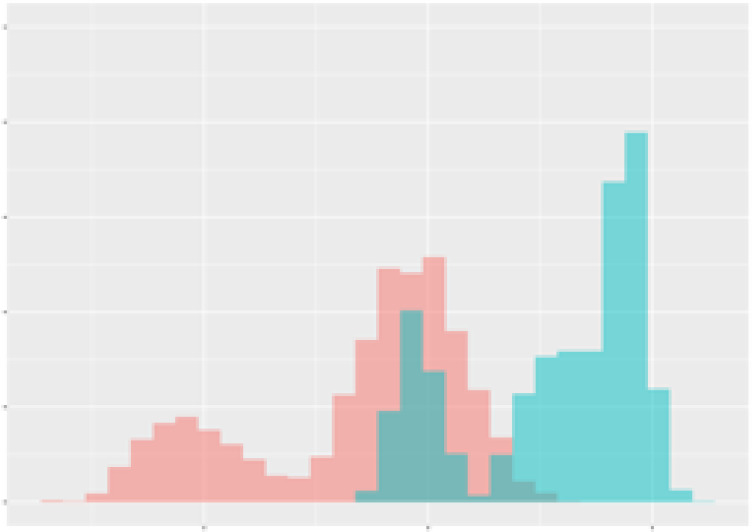 83.00 ± 12.23 [%]	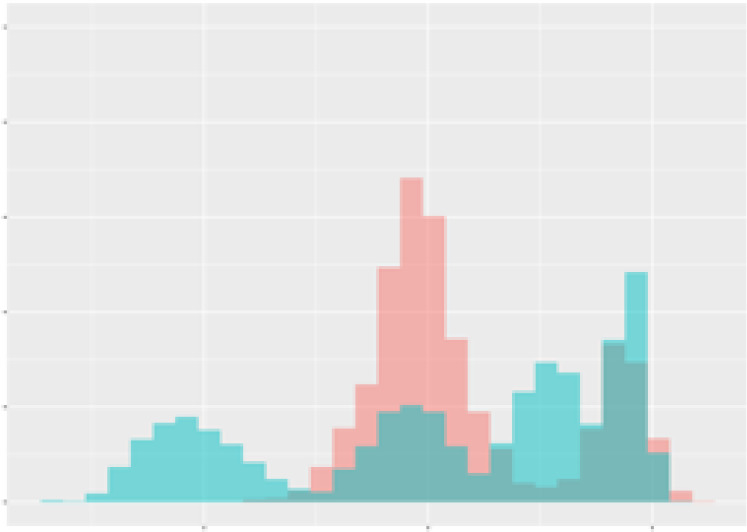 43.83 ± 16.76 [%]	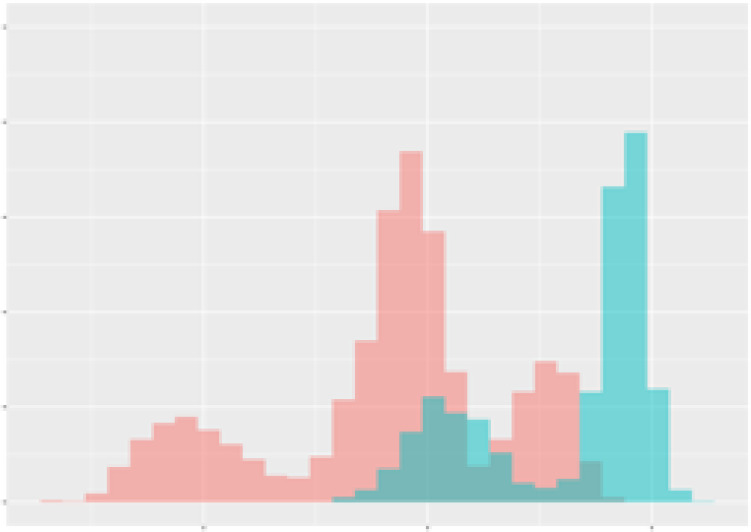 73.93 ± 16.01 [%] *)
**CO2** **[ppm]** **x=[490, 910]** **y=[0,400]**	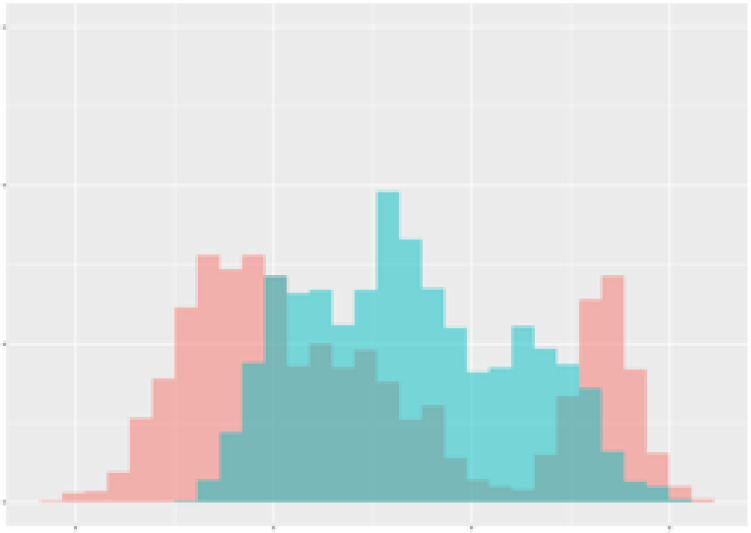 83.93 ± 9.98 [%]	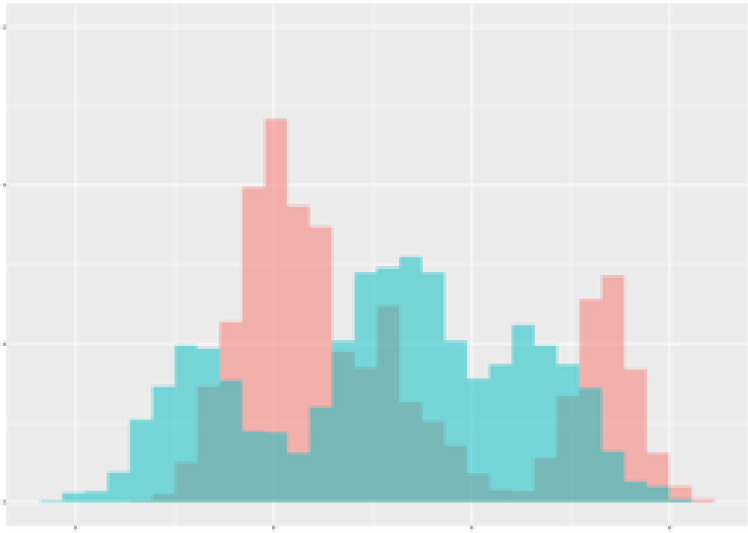 70.08 ± 11.78 [%]	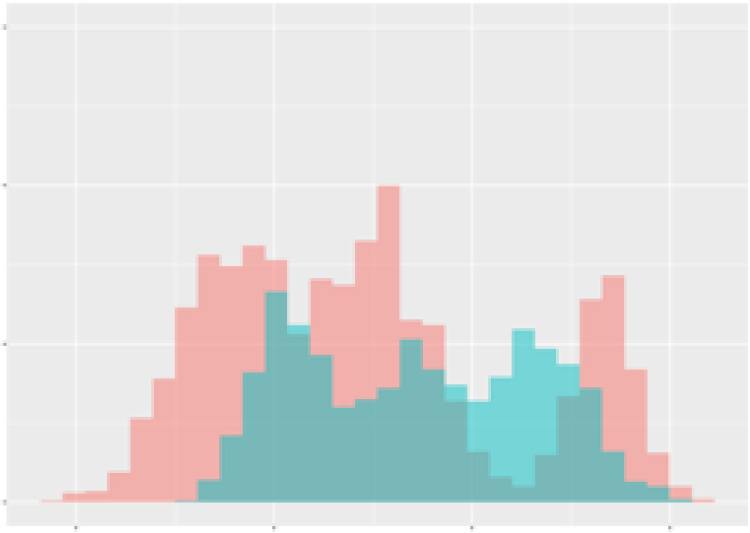 63.93 ± 14.23 [%]	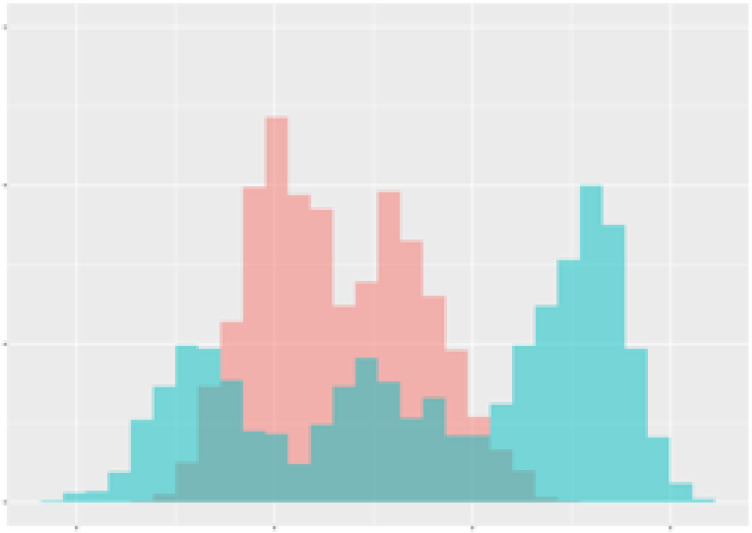 60.20 ± 14.66 [%]	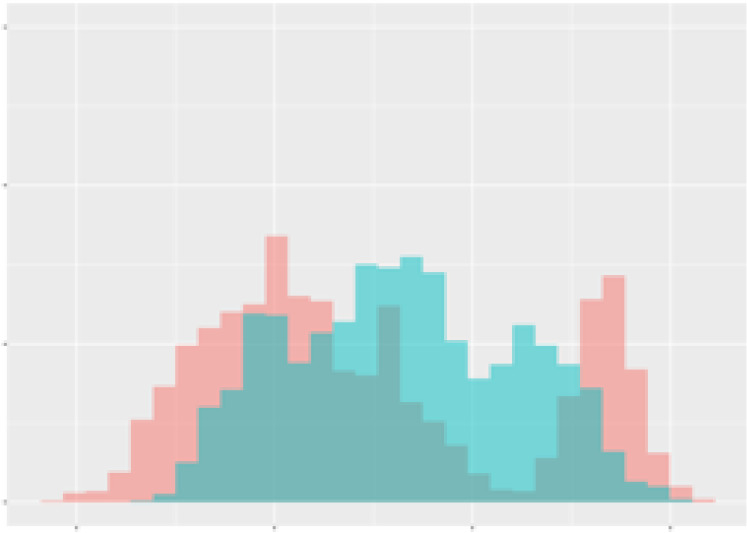 83.03 ± 11.06 [%]	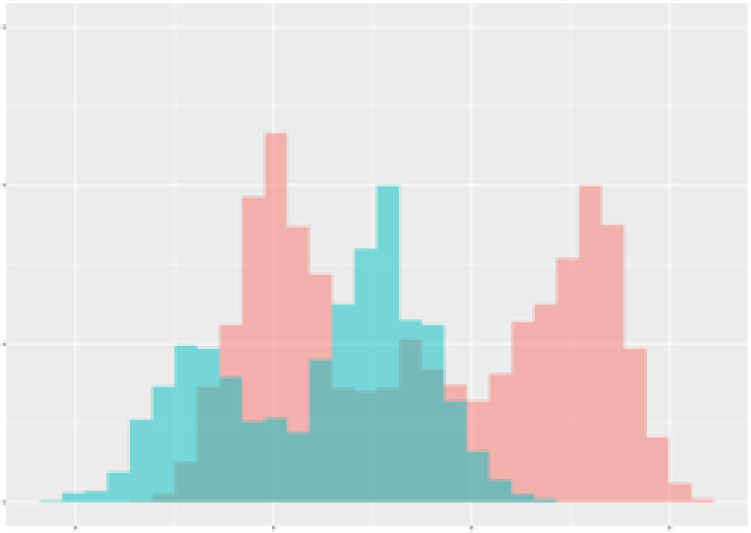 71.20 ± 13.28 [%]
**Radiation** **[PPFD]** **x=[30, 920]** **y=[0,500]**	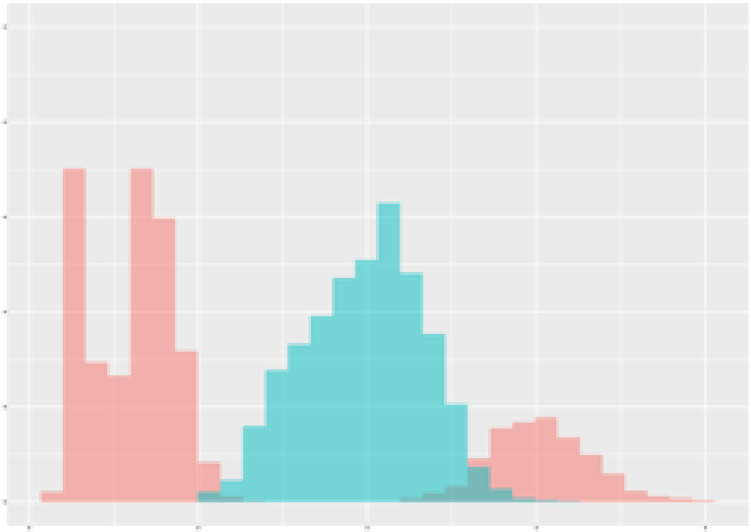 97.18 ± 2.37 [%] *)	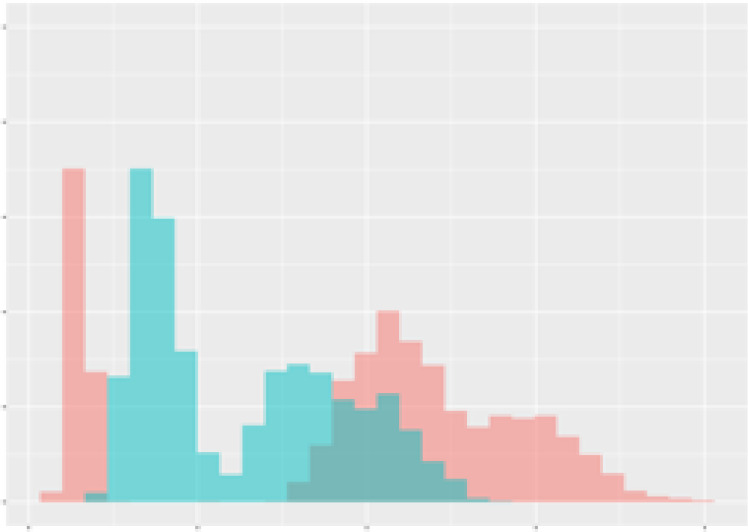 94.68 ± 3.26 [%] *)	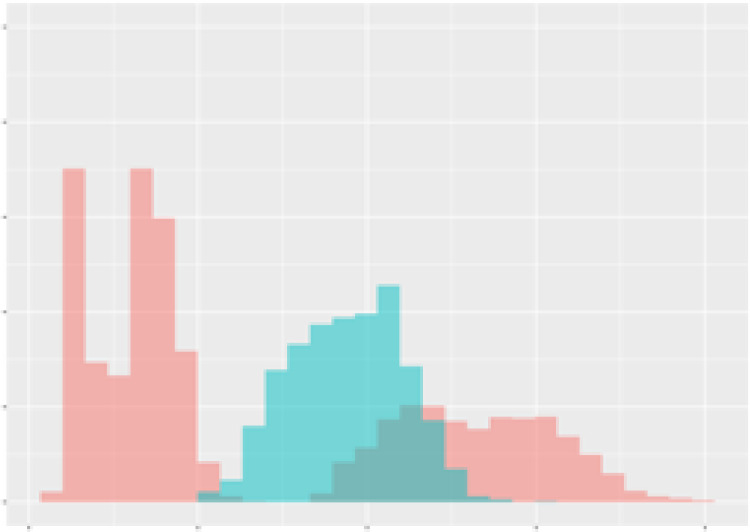 94.38 ± 3.69 [%] *)	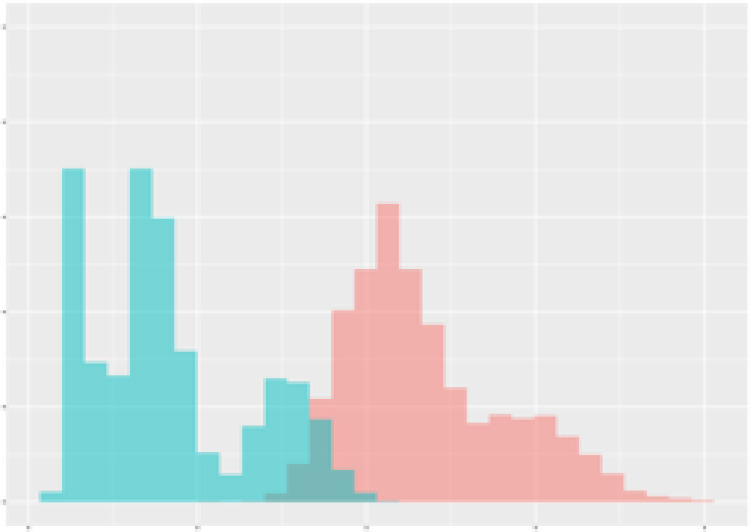 81.78 ± 12.73 [%] *)	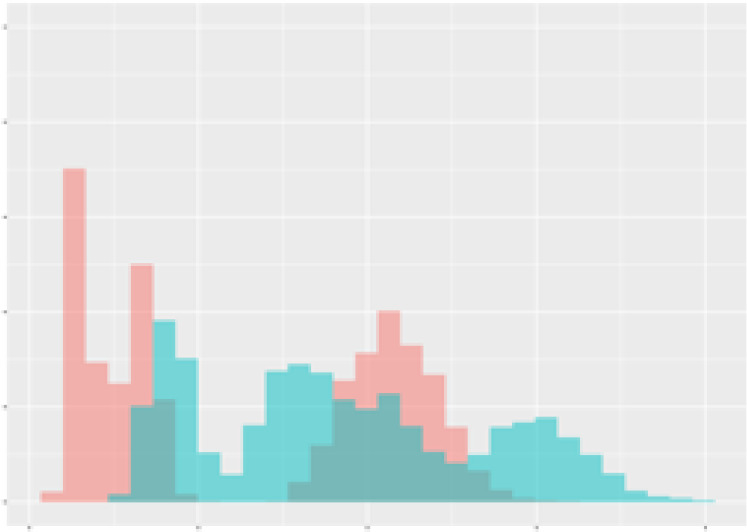 57.63 ± 16.17 [%]	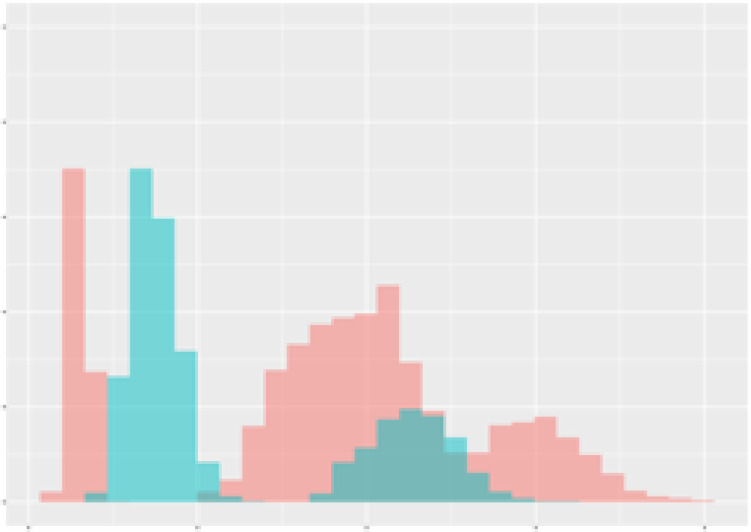 59.78 ± 15.94 [%]

Histograms of the mean values of augmented time series alongside with the corresponding labelling of the secondary metabolite content. x and y represent the ranges of x-axis and y-axis of the corresponding channel respectively. Class 0 is depicted in red, whereas Class 1 is depicted in green. The percentage numbers show the accuracy of the corresponding classification task. Group 1: beta carotene, lutein, lycopene. Group 2: caffeic acid derivates, coumaric acid hexoside, ferulic acid hexoside, quercetin. *) indicates channels with the highest impact.

**Table 10:** Greenhouse parameter channels against total secondary metabolites.

Channel	Total carotenoids	Total phenolic acids	Total flavonoids
**Temperature** **[°C]** **x=[18, 28]** **y=[0, 500]**	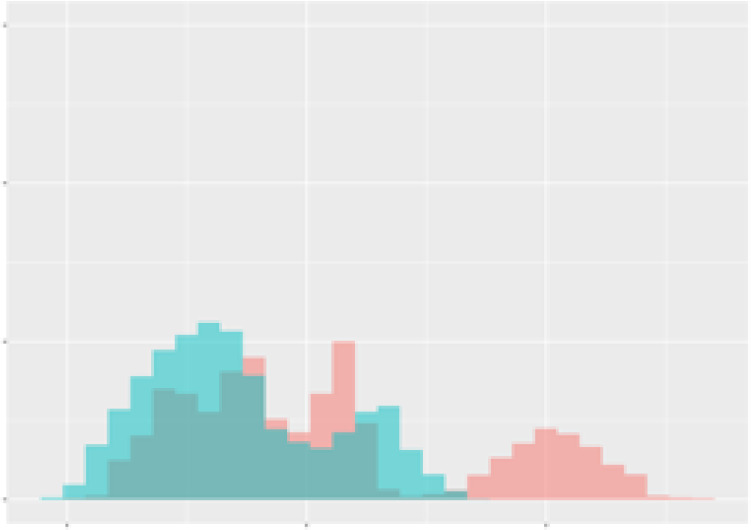 72.7 ± 13.47 [%]	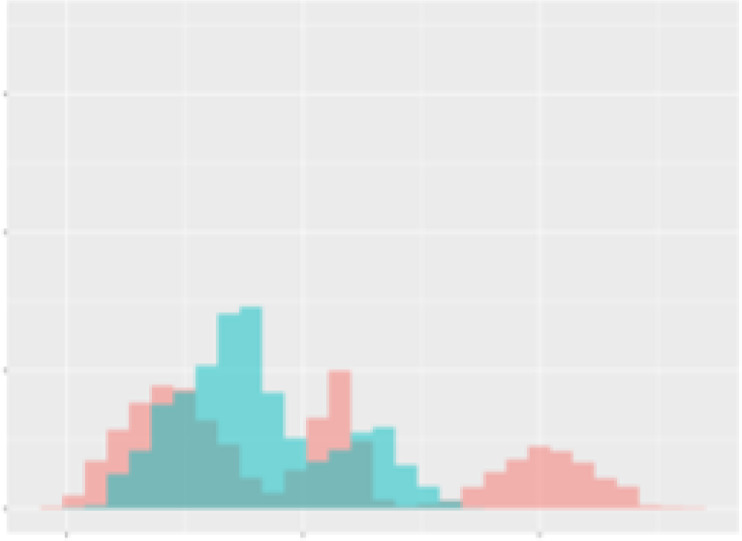 82.05 ± 9.24 [%]	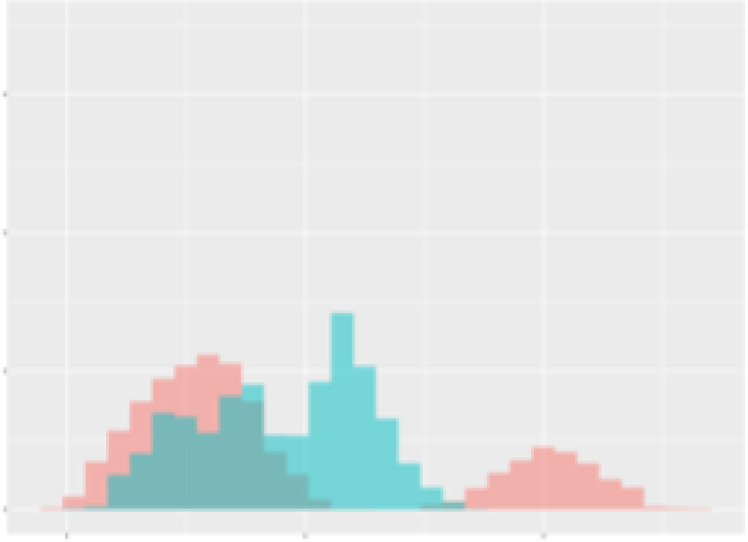 75.28 ± 11.26 [%]
**Photosynthesis** **[µmol CO2/qm s]** **x=[-16, 1.5]** **y=[0,1200]**	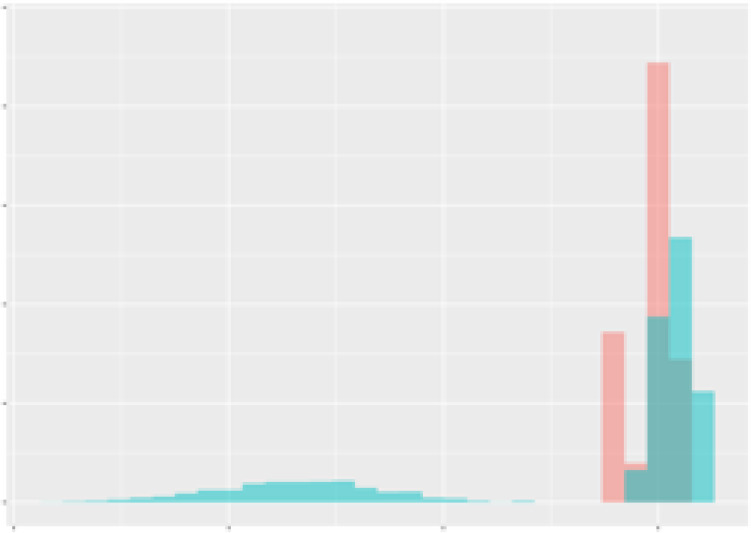 37.35 ± 18.09 [%]	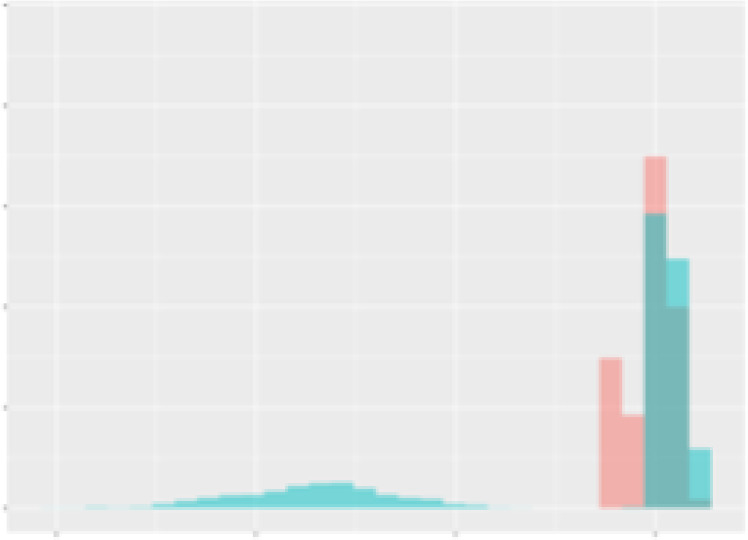 38.13 ± 15.54 [%]	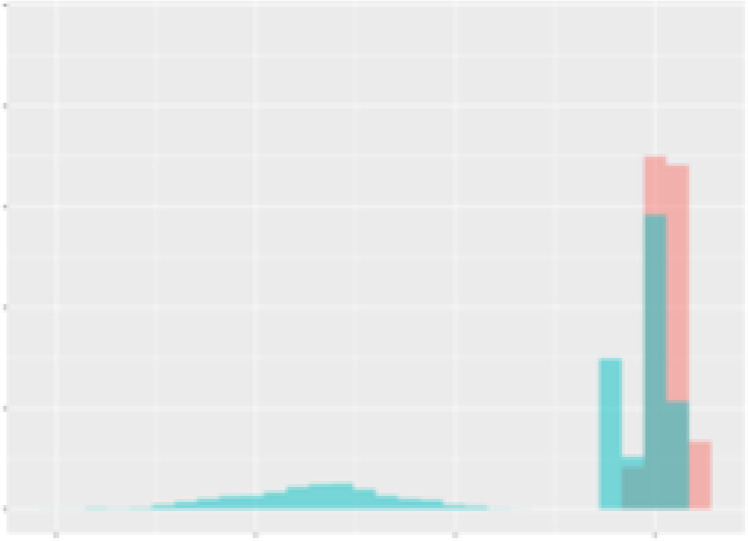 66.5 ± 15.86 [%]
**Transpiration** **[mg H2O/qm s]** **x=[3, 15]** **y=[0,750]**	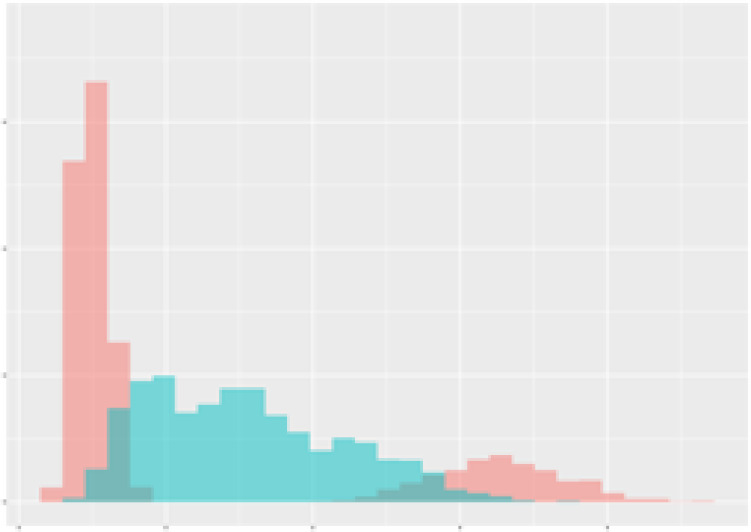 96.75 ± 3.03 [%] *)	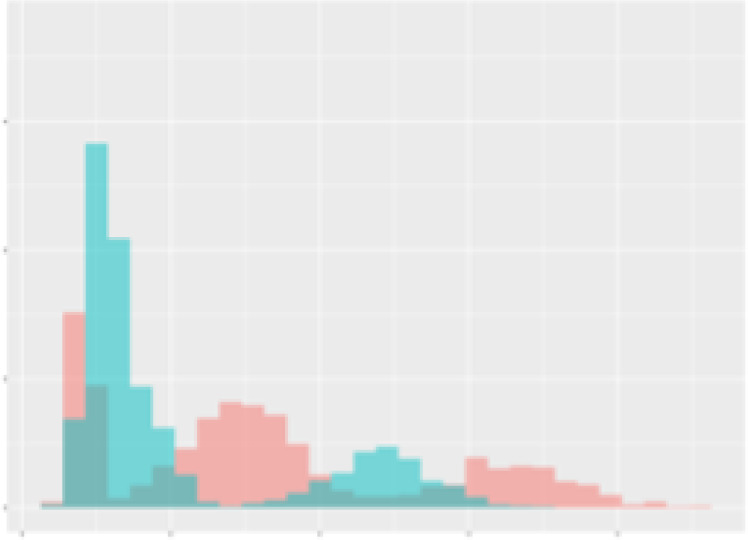 76.825 ± 13.29 [%]	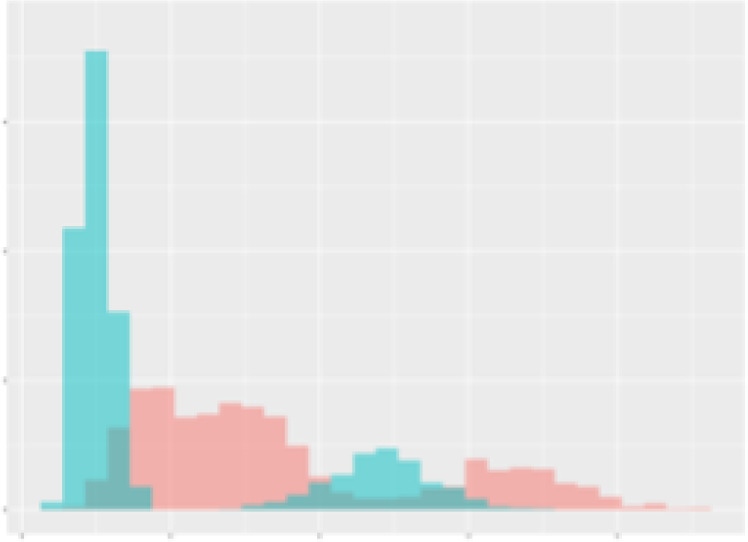 74.575 ± 14.72 [%]
**Humidity** **[%]** **x=[60, 95]** **y=[0,500]**	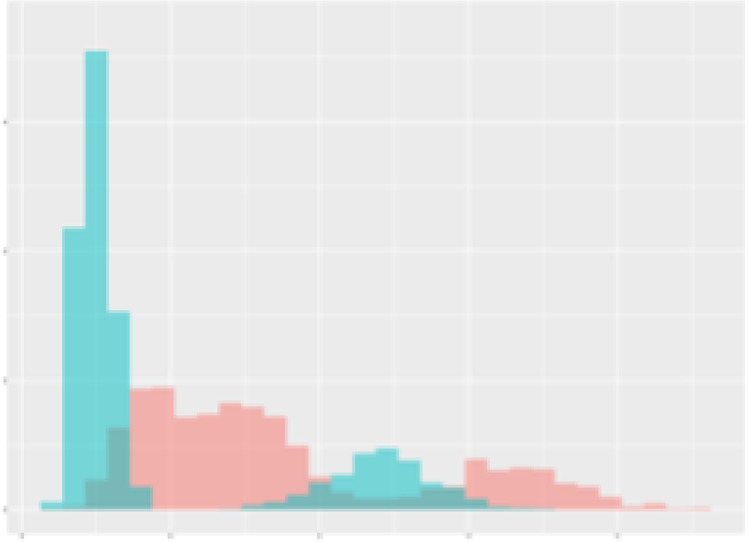 85.00 ± 11.41 [%]	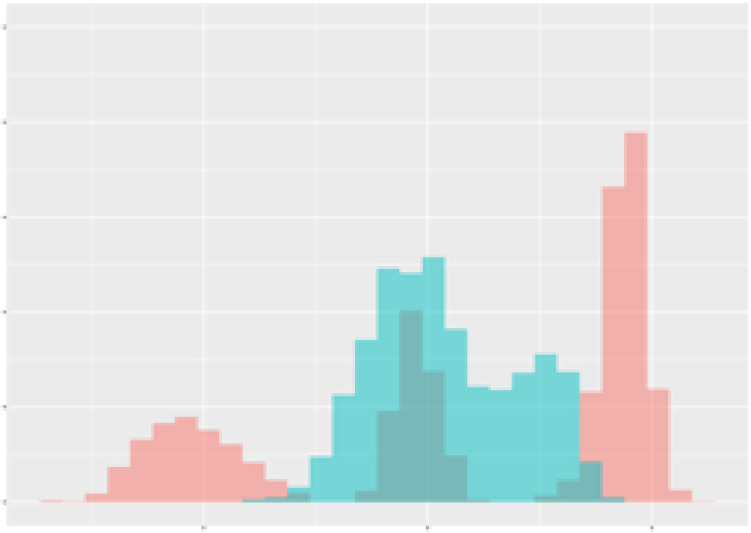 90.725 ± 9.048 [%] *)	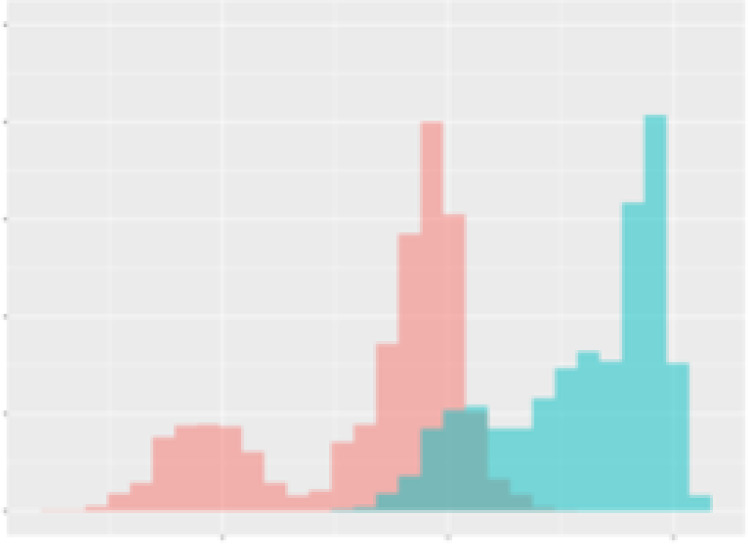 81.15 ± 12.39 [%] *)
**CO2** **[ppm]** **x=[490, 910]** **y=[0,400]**	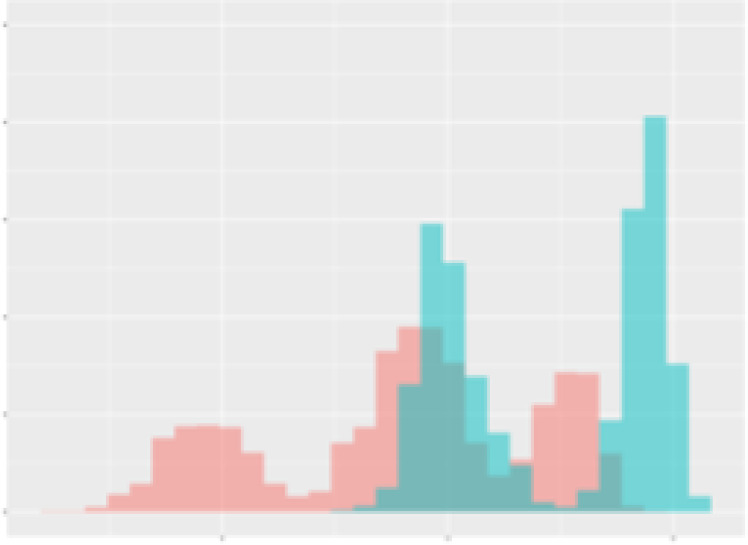 83.93 ± 9.98 [%]	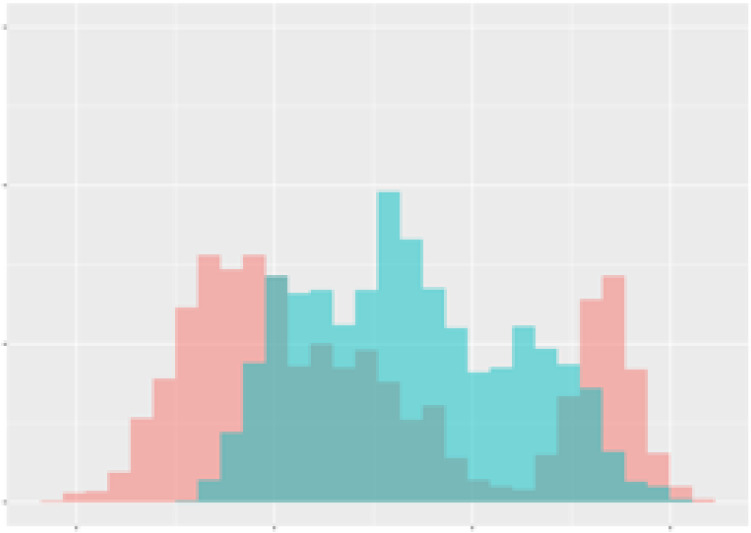 67.325 ± 11.46 [%]	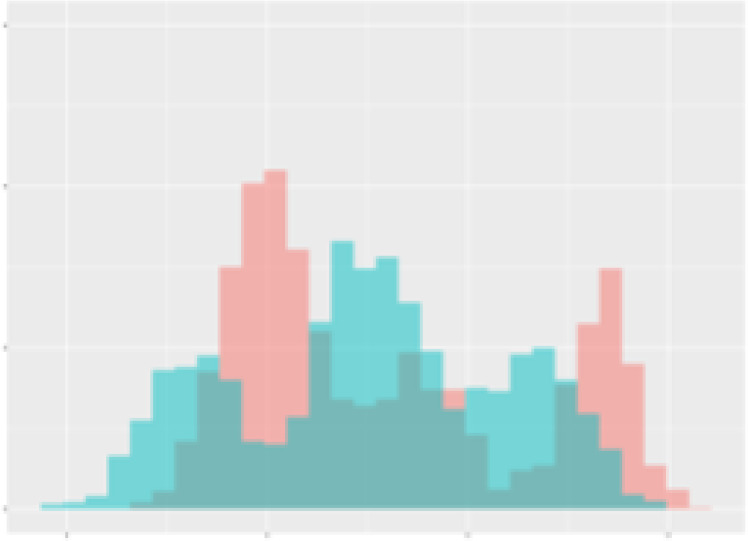 55.675 ± 13.4 [%]
**Radiation** **[PPFD]** **x=[30, 920]** **y=[0,500]**	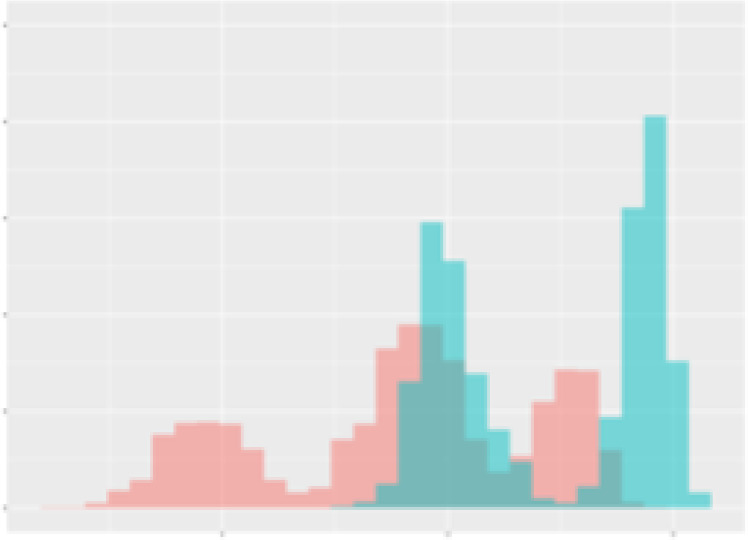 97.175 ± 2.37 [%] *)	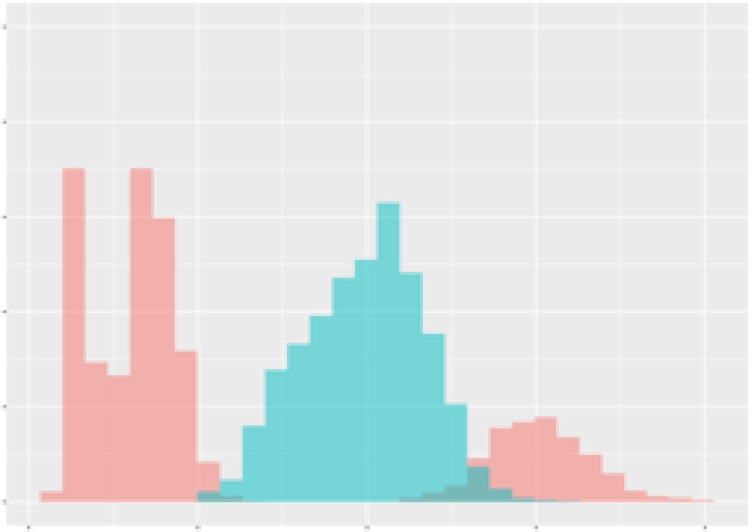 87.55 ± 7.355 [%] *)	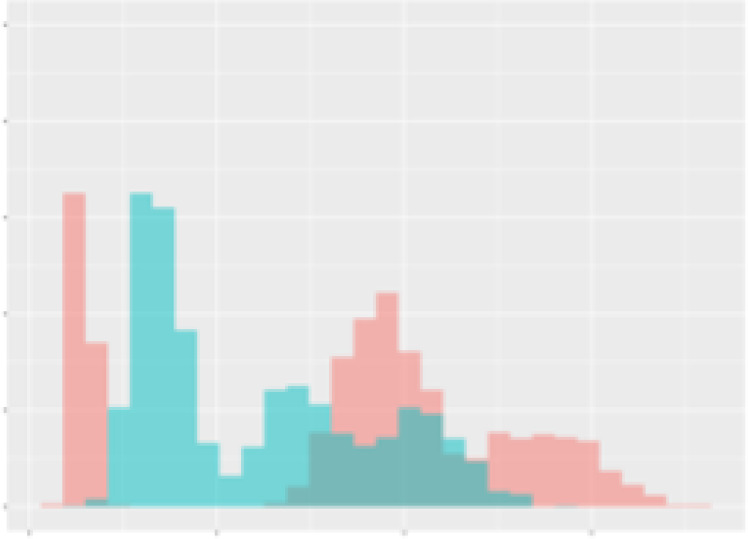 80.325 ± 12.96 [%] *)

Histograms of the mean values of augmented time series alongside with the corresponding labelling of the total secondary metabolite content. x and y represent the ranges of x-axis and y-axis of the corresponding channel respectively. Class 0 is depicted in red, whereas Class 1 is depicted in green. The percentage numbers show the accuracy of the corresponding classification task. *) indicates channels with the highest impact.

Generally, other studies indicate that carotenoids and phenolic substances accumulate more strongly in tomatoes with increasing light intensity (Slimestad and Verheul, 2005; Formisano et al., 2021). This suggests that light intensity has a significant influence on secondary metabolites. However, considering flavonoids such as naringenin and phloretin diglucoside, as well as carotenoids (lycopene, β-carotene, and lutein and thus total carotenoids), light intensity alone is insufficient as an explanatory variable, as transpiration, in addition to light intensity, also influences the synthesis of these secondary metabolites (**Table 9**).

As such, radiation levels between 396.0 μmol/m^2^s and 511.2 μmol/m^2^s were observed as optimal for the contents of beta carotene, lutein, and lycopene, while lower ranges from 229.4 μmol/m^2^s to 431.2 μmol/m^2^s and from 35.76 μmol/m^2^s to 262.28 μmol/m^2^s are beneficial for naringenin and phloretin contents, respectively (**Table 11**). Simultaneously, transpiration rates ranging from 4.4 mg H_2_O/m^2^s to 7.47 mg H_2_O/m^2^s over a period of three weeks were observed as optimal for the contents of beta carotene, lutein, and lycopene, while slightly different ranges from 4.71 to 6.47 mg H_2_O/m^2^s and from 3.04 to 4.26 mg H_2_O/m^2^s are optimal for naringenin and phloretin contents, respectively.

**Table 11:** Optimal ranges of the greenhouse channels for the individual secondary metabolites content.

	Group 1	Group 2	Naringenin	Phloretin	Coumaroylquinic acids	Caffeoylquinic acid derivates
temperature [°C]	–	20.94 – 21.53	–	–	–	–
photosynthesis [µmol CO_2_/m^2^ s]	–	0.39 – 1.21	–	–	0.39 – 1.21	–
transpiration [mg H_2_O/m^2^ s]	4.40 – 7.47	–	4.71 – 6.47	3.04 – 4.26	–	–
humidity [%]	–	–	–	–	–	86.14 – 91.29
CO_2_ [ppm]	–	–	–	–	–	–
radiation [PPFD]	396.0 – 511.2	250.0 – 375.2	229.4 – 431.2	35.76 – 262.28	–	–

Non-presence ( - ) indicates that no direct relationship of the channel was observed. Group 1: beta carotene, lutein, lycopene. Group 2: caffeic acid derivates, coumaric acid hexocide, ferulic acid hexocide, quercetin.

Less surprisingly, the CO_2_-concentration has no influence on either carotenoids or the phenolic compounds. The same has also been proven in other studies (Boufeldja et al., 2023; Oliva-Ruiz et al., 2023; Pimenta et al., 2023). However, caffeoylquinic acid derivates are the only ones which are solely explained by relative humidity (**Table 9**). A mean relative humidity between 86.14% and 91.29% over 3 weeks yielded the best results for the accumulation of these phenolic acids in tomatoes. A correlation between relative humidity and phenolic acids has thus been demonstrated for the first time.

It is interesting that a mean photosynthesis rates between 0.39 μmol CO_2_/m^2^s and 1.21 μmol CO_2_/m^2^s over three weeks were also crucial for the optimal accumulation of phenolic acids such as caffeic acid derivates, coumaric acid hexoside, ferulic acid hexoside and coumaroylquinic acids as well as for quercetin as flavonoid. Simultaneously, the optimal temperature range between 20.94 and 21.53°C and a PPFD from 250.0 to 375.2 μmol/m^2^s was classified as beneficial to synthesize the aforementioned compounds with the exception of coumaroylquinic acids, which were solely dependent on photosynthesis (**Table 11**). Other studies have also found that this temperature range can be seen as optimum for the accumulation of phenolic compounds (Toor et al., 2006; Krumbein et al., 2012).

While relative humidity only plays a subordinate role in the synthesis of the individual phenolic compounds, the situation is quite different for total phenolic acids and total flavonoids (**Table 10**).

In addition to an optimum light intensity for the total phenolic acids between 129.35 - 274.34 μmol/m^2^s and for the total flavonoids between 31.24 - 249.31 μmol/m^2^s, the optimum relative humidity levels are between 83.45 - 91.29% and 87.13 - 91.29%, respectively (**Table 12**). This proves for the first time that relative humidity plays a decisive role in the optimization of the individual substance classes in tomatoes. It was furthermore demonstrated for the first time that, in addition to the optimal light intensity (396.0 – 511.2 μmol/m^2^s), the optimal transpiration rates (4.40 – 7.47 mg H_2_O/m^2^s) during a three-week ripening process are crucial for the accumulation of total carotenoids (**Tables 10**, **12**).

**Table 12:** Optimal ranges of the greenhouse channels for the total secondary metabolites content.

	Total carotenoids	Total phenolic acids	Total flavonoids
temperature [°C]	–	–	–
photosynthesis [µmol CO_2_/qm s]	–	–	–
transpiration [mg H2O/qm s]	4.40 – 7.47	–	–
humidity [%]	–	83.45 – 91.29	87.13 – 91.29
CO_2_ [ppm]	–	–	–
radiation [PPFD]	396.0 – 511.2	129.35 – 274.34	31.24 – 249.31

Non-presence ( - ) indicates that no direct relationship of the channel was observed.

**Figure 2:**
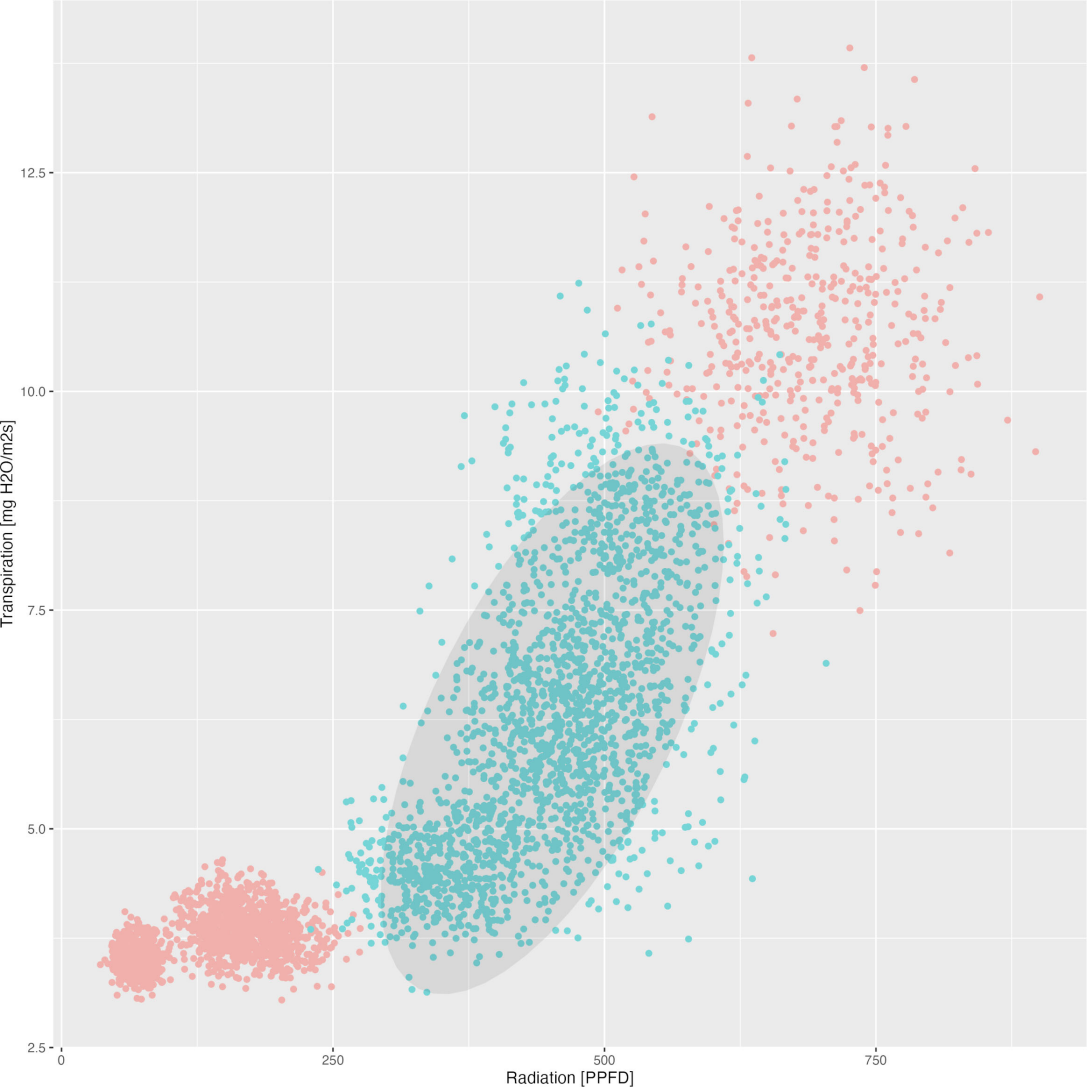
Dependency between mean radiation and mean transpiration in relation to carotenoid content. Green points describe class 1 with high carotenoid content. Grey ellipse contains 90% of observed (augmented) points of corresponding time-series.

Finally, as an example consider the **Figure 2** which demonstrates the observed dependency between mean radiation and mean transpiration (over 3 weeks) in context of the total carotenoid contents. The data points are plotted to show how these two environmental factors correlate with carotenoid production. An ellipse is drawn to encompass 90% of the observed data points, representing the optimal range of radiation and transpiration conditions that are associated with higher total carotenoid contents. This visual representation provides an approach for adjusting greenhouse conditions to enhance carotenoid production in tomatoes.

## 4 Conclusion and outlook

In this study, we successfully integrated and analyzed time series greenhouse data to classify secondary metabolites content using data-driven techniques. The application of moving block bootstrap augmented the raw dataset, allowing for reliable classification and accuracy evaluation through eight-fold cross-validation. The identification of the most impactful channels provided important explanatory information for the secondary metabolite content prediction. Furthermore, the derivation of optimal channel ranges through presented class histogram analysis offers a practical approach into the achievement of high secondary metabolite contents. With this technique we provide the first evidence that the impact of a single climate factor on secondary metabolites in tomato fruits should not be considered in isolation, but rather, all climatic factors including radiation, temperature, relative humidity and CO_2_-concentration as well as plant responses such as transpiration and photosynthesis during a growth period must be taken into account to predict the maximum accumulation of individual phenolic compounds and carotenoids in tomatoes. In this context, we were able to demonstrate for the first time the influence of relative humidity as well as photosynthesis and transpiration rates on the accumulation of secondary metabolites in tomatoes. With these results, researchers should be encouraged to push for further trials to maximize the accumulation of secondary metabolites in different fruits and vegetables with the application of multiple variables. Our results have laid the headstone to help growers target their climate controls to maximize the health-promoting phytochemicals in tomatoes.

## Data Availability

The datasets presented in this study can be found in online repositories. The names of the repository/repositories and accession number(s) can be found below: https://github.com/acsd-tu-chemnitz/secondary_metabolites.
